# Rapid Fabrication of Round Flexdym Microchannels via Direct PDMS (Thermoset)-to-Flexdym (Thermoplastic) Thermal Replication

**DOI:** 10.3390/polym18192387

**Published:** 2026-09-30

**Authors:** Md Emamul Kabir, Sonali Garg, Aldrian Muhaxheri, Paras N. Prasad, Kwang W. Oh

**Affiliations:** 1 SMALL (Sensors and MicroActuators Learning Lab), Department of Electrical and Computer Engineering, University at Buffalo, The State University of New York, Buffalo, NY 14260, USA; mdemamul@buffalo.edu (M.E.K.);; 2 Department of Chemistry, University at Buffalo, The State University of New York, Buffalo, NY 14260, USA; sonali@buffalo.edu (S.G.);; 3 The Institute for Lasers, Photonics, and Biophotonics, University at Buffalo, The State University of New York, Buffalo, NY 14260, USA; 4 Department of Biomedical Engineering, University at Buffalo, The State University of New York, Buffalo, NY 14260, USA

**Keywords:** Flexdym, PDMS, round microchannels, thermoplastic microfluidics, thermal air expansion, thermal replication, PDMS molding, microvalve

## Abstract

Round microchannels provide physiologically relevant flow environments and improved fluidic performance compared with conventional rectangular geometries; however, their scalable fabrication in potentially biocompatible thermoplastic substrates remains challenging. Here, we present a rapid and low-cost PDMS-to-Flexdym thermal replication strategy to directly transform round microchannel architectures from a reusable compliant thermoset PDMS mold into a thermoplastic microfluidic platform. Round PDMS convex molds generated by thermal air expansion followed by PDMS double casting were used for heat-assisted conformal replication to transform spatially varying complex round microchannels into Flexdym. The method successfully transferred complex serpentine, branched, and interconnected channel architectures while preserving continuous flow paths and curved geometries. Temperature-dependent framework and experimentation revealed improved vertical feature recovery with increasing embossing temperature, while lateral dimensions remained highly conserved. Across representative channel widths of ~37–249 µm, replicated Flexdym channels achieved width and height transfer ratios of approximately 99–103% and 93–105%, respectively. Sequential dimensional analysis among concave PDMS, convex PDMS, and Flexdym replicas showed no statistically significant differences in channel width or height (*p* > 0.05), confirming high geometric fidelity throughout the replication process. AFM, SEM, and stylus profilometry results further verified the preservation of surface characteristics, rounded cross-sectional morphology, and reproducible channel profiles, with Flexdym surface roughness reduced from ~84.1 nm (PDMS mold) to ~25.1 nm in Flexdym after replication. Finally, integration of the replicated round channels into a multilayer microvalve demonstrated functional fluidic control through syringe-assisted compressed air actuation, supported by two-phase moving-mesh simulation and analytical air compression modeling. This strategy provides a practical route for translating PDMS-based round microchannel prototyping into scalable, more biocompatible thermoplastic microfluidic platforms for building next-generation microphysiological systems and bioengineering applications.

## 1. Introduction

Performance of microfluidic systems is strongly governed by channel geometry because the cross-sectional shape determines the flow path that influences velocity profile, pressure, and wall-shear-stress distributions, which affect mixing, control, surface interactions, convection, and diffusion [1,2,3,4]. Rectangular channels produced by planar lithography and digital manufacturing remain the standard in current practice [5]. However, many biologically relevant fluidic environments, including blood perfusion in vessels and capillary-scale structures, are more accurately represented by round or nearly circular microchannels rather than sharp-edged rectangular cross-sections [2,6,7,8,9]. For this reason, round microchannels have been explored to better mimic three-dimensional physiological flow as well as microenvironments and to study vascular transport, endothelial mechanobiology, and vessel-associated pathophysiology [6,10,11]. In addition, round microchannels also help reduce stagnant “corner” regions and dead volume, mitigate particle or cell trapping at sharp edges, and improve performance in applications such as single-cell handling, flow control, droplet generation, nanoparticle synthesis, cellular interactions, and organ-on-a-chip platforms [12,13,14]. Despite their advantages, round microchannels remain considerably more challenging to fabricate than conventional planar microchannels.

Following the literature, approaches include photoresist reflow, mechanical micromilling combined with replica molding, sacrificial or removable templates, thermally or pneumatically induced polymer reshaping, and, more recently, additive-manufacturing and membrane-inflation strategies [7,15,16,17,18,19,20]. These methods provide different levels of geometric control but involve trade-offs in master fabrication, process complexity, dimensional tunability, post-processing, or compatibility with manufacturing-oriented substrates [21]. Specifically, the challenges come through while making complex, spatially varying round microchannel architectures in thermoplastic substrates [22,23]. Indeed, a compliant PDMS (polydimethylsiloxane) master has been successfully demonstrated for rectangular cross-sectional shape replication via hot embossing [23]. However, making round microchannel masters and, therefore, replicating them on engineering-friendly thermoplastic substrates, along with rapid, low-cost prototyping for microfluidic applications, has diverse engineering hurdles to tackle, from the choice of material and its properties to process complexity, cost, and process time.

Among the materials used for prototypes of such microsystems, polydimethylsiloxane (PDMS) has dominated academic research in microfluidics for over two decades because it is optically transparent, inexpensive, biocompatible, and gas-permeable [24], and it is easily molded by soft lithography into complex architectures [25,26,27]. Despite these advantages, PDMS presents several important limitations for translation beyond laboratory prototyping. It is hydrophobic, and it adsorbs small hydrophobic molecules and biomolecules, thereby potentially compromising preclinical safety assessment [28,29,30], swelling in many organic solvents [31], and undergoing hydrophobic recovery after plasma activation while also permitting the leaching of uncrosslinked oligomers. In addition, PDMS is a thermoset elastomer that is poorly compatible with high-throughput industrial processes such as injection molding and roll-to-roll manufacturing, unlike thermoplastic polymers, which limits its direct translation from prototyping to scale-up, also because of its relatively slow casting cycle [32].

To tackle this, the microfluidics field has increasingly shifted toward thermoplastics such as cyclic olefin copolymer, poly(methyl methacrylate), and polycarbonate because they offer transparency and chemical and heat resistance, and they are readily fabricated by hot embossing, injection molding, laser micromachining, and micro-milling for high-volume manufacturing [32]. A particularly attractive low-cost material in this context is Flexdym, a styrene-ethylene-butylene-styrene (SEBS)-based thermoplastic elastomer that has emerged as a next-generation microfluidic material to address major limitations of PDMS, including hydrophobic compound absorption, low chemical inertness, multistep bonding processes, and limited manufacturability at large scale (Flexdym™, Eden Tech Microfluidics, Paris, France) [33]. It can be molded in minutes and then reversibly or irreversibly bonded rapidly both at room temperature and upon heating to glass or thermoplastic substrates through simple conformal contact, without plasma or adhesives [34,35]. Recent studies further indicate that Flexdym and related thermoplastic elastomers can exhibit improved chemical resistance, lower adsorption of small hydrophobic molecules and proteins, no oligomer leaching, stable hydrophilicity, cytocompatibility, flexibility, gas permeability, as well as enable non-destructive tissue recovery compared with PDMS, making them attractive for long-term cell culture, adhesion, spreading, proliferation and migration, point-of-care diagnostics, cell manipulation, protein detection, wearable electronics and ultra-stretchable microfluidics. In addition, Flexdym will be potentially useful for next-generation microphysiological systems such as organ-on-a-chip, microfluidic bioreactors that require biocompatibility (ISO 10993 and USP Class VI-compliant), reproducibility, transparency, scalability, and more reliable control of drug exposure for in vitro testing than PDMS-based devices typically provide [3,33,34,36,37,38,39,40]. However, reliable, low-cost and rapid replication of round microchannels in Flexdym for microfluidic applications is promising and unexplored, having only been attempted in an online technical note by Elveflow with large, fixed-diameter metal wires [41].

To address this unmet need, we present a rapid, simple, reliable and low-cost strategy for directly replicating round-microchannel architectures from a reusable PDMS (thermoset) master into Flexdym (thermoplastic). In this approach, round PDMS microchannels are first generated by thermal air expansion [2,16,42]. The convex counterparts of round PDMS microchannels act as a compliant master mold used for replicating the PDMS-encoded pattern into Flexdym using a laboratory-made screw-ram glass contact system. Unlike conventional thermoplastic fabrication routes that typically favor planar rectangular or trapezoidal geometries, this method enables replication of smooth, curved, complex, spatially varying microchannel features into Flexdym in one shot while preserving high structural fidelity. This study establishes and quantitatively validates a compliant-mold thermal replication process into Flexdym, including dimensional fidelity, cross-sectional shape preservation, mold reusability, and fluidic performance of the fabricated microvalve demonstrated as a proof-of-concept. This work will mark a potential pathway for future biological, microreactor, and organ-on-a-chip applications.

## 2. Materials and Methods

### 2.1. PDMS Convex Mold Fabrication

A silicon wafer (UniversityWafer, Inc., South Boston, MA, USA) containing patterned negative SU-8 (Kayaku Advanced Materials, Inc., Westborough, MA, USA) photoresist features of 73.54 ± 0.32 µm was first fabricated by standard photolithography (Mask aligner, Karl Suss MA6/BA6, University at Buffalo) to serve as the master mold SU-8/silicon for the thermal air expansion technique (mask design, Supplementary Figure S1). PDMS prepolymer and curing (Dow Inc., Midland, MI, USA) agent were mixed at a 10:1 (*w*/*w*) ratio, degassed, and poured into a 3D-printed (X1 Carbon, Bambu Lab, Shenzhen, China) clear polycarbonate frame (60 mm × 50 mm × 4 mm, wall width 3 mm) attached to an HMDS-coated glass substrate. After 14 min of partial curing on a hotplate, the PDMS layer was placed face-down onto the SU-8/silicon master and heated at 90 °C for 1 h using the thermal air expansion method to generate curved concave PDMS microchannels (CC), as described in our reported literature [42]. After curing, the PDMS replica was peeled off from the SU-8/silicon master. A convex PDMS mold (CV) was fabricated by a PDMS double-casting process from the CC [42,43]. First, CC was plasma-treated for 30 s at medium RF level (Plasma cleaner PDC-32G, Harrick plasma, Ithaca, NY, USA), passivated with methanol for 30 min, and dried at 80 °C for 30 min in the oven. The treated CC was attached to a similar 3D-printed polycarbonate frame, followed by pouring uncured PDMS (PDMS prepolymer and curing agent mixed at a 10:1 (*w*/*w*) ratio) on it and curing in the oven at 80 °C for 2 h. After curing, the replicated PDMS was peeled off to obtain the CV, the master mold used for the thermal replication process (Supplementary Figure S2).

### 2.2. Flexdym Round Channel Fabrication

**Figure 1:**
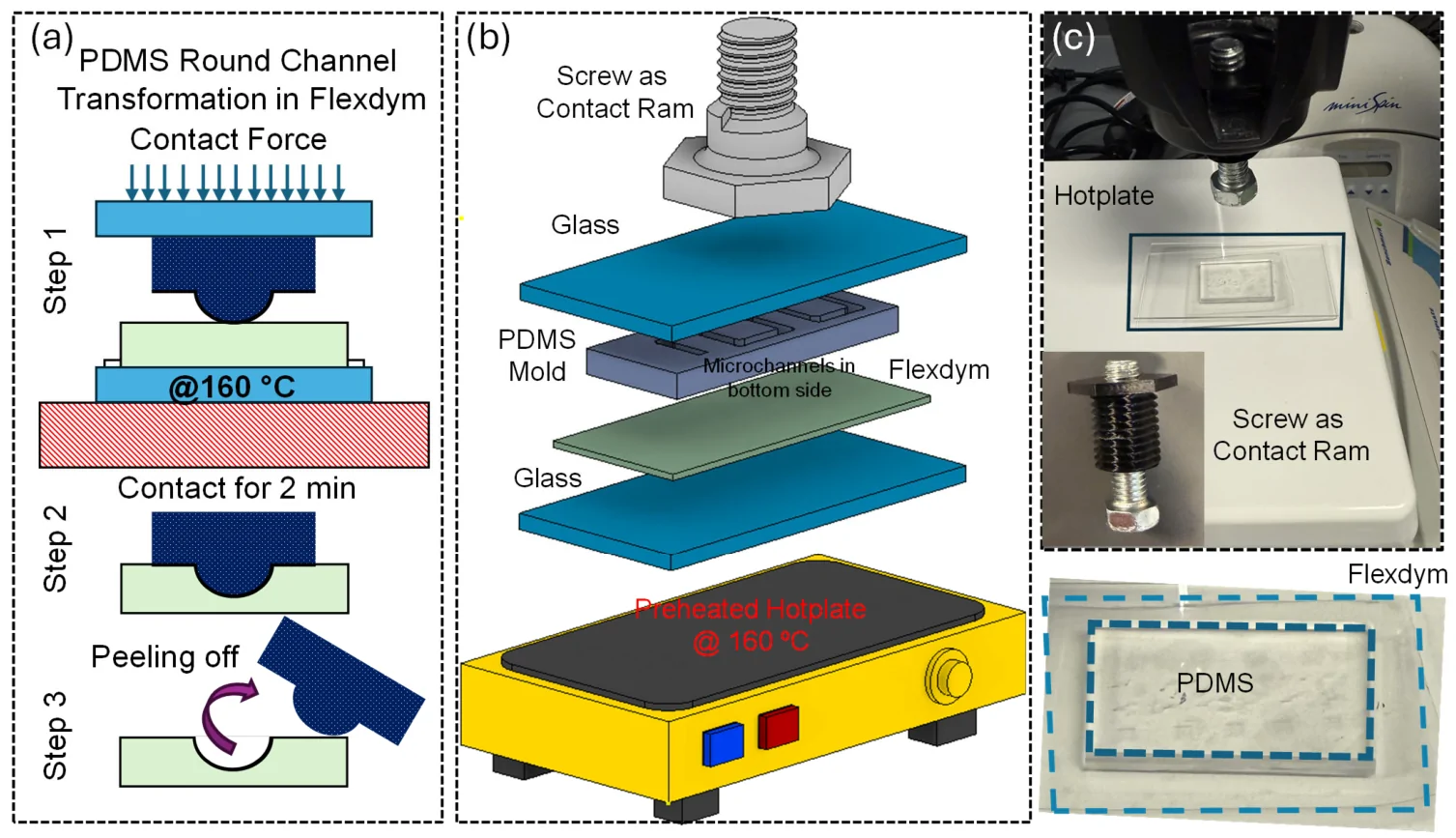
(**a**) Schematic illustration of the Flexdym molding and demolding process. Under heat and conformal contact, the Flexdym layer conforms to the raised microfeatures of the PDMS convex mold (Supplementary Figure S2) on a preheated hotplate, followed by peeling/demolding to reveal the replicated microchannel features. (**b**) Exploded schematic of the thermal replication setup, showing the layer stack: bottom glass support, Flexdym sheet, PDMS mold, top glass plate, and a screw-driven contact system to apply uniform contact (Supplementary Figure S2). (**c**) Photographs of the experimental setup, including the hotplate, screw-ram, assembled glass–Flexdym–PDMS stack, and the PDMS mold used for feature transfer.

The thermal replication process begins with placing a glass plate on the hotplate. A Flexdym sheet 250 µm thick (Eden Tech Microfluidics, Paris, France) was then cut with scissors to a size slightly larger than the required device dimensions. After peeling off the protective plastic film, the exposed surface was placed face up on the hotplate, and the convex PDMS mold was placed face down on the Flexdym. A second glass plate was placed above the PDMS to help distribute contact force evenly with the screw-ram glass contact system. The full assembly was preheated at 160 °C for approximately 5 min so that the Flexdym softened and conformed more effectively to the convex PDMS mold. After preheating, upon conformal contact for 3 min using the modified screw-ram glass contact system attached to the hand lever of inexpensive Rotary Tool WorkStation stand (Dremel 220-01, Robert Bosch Tool Corporation, IL, USA) as shown in Figure 1 (Supplementary Figure S3). After holding it for 3 min, the assembly was removed from the hotplate and allowed to cool at room temperature for approximately 2 min. Then, the glass plates were carefully removed. This allowed temperature-dependent round microchannel replication into Flexdym and preserved the replicated features (F_d_). Finally, the Flexdym was peeled off the PDMS mold and inspected. Width was measured with an upright optical microscope (Unitron EXAMET-5, Model 14350, Commack, NY, USA) and step height using a Veeco Dektak 150 stylus profilometer (Plainview, NY, USA) (Electrical and Computer Engineering Cleanroom, University at Buffalo) to characterize the replication quality in PDMS-to-Flexdym. To make a full round microchannel, the complementary semi-round Flexdym channel layers were first oxygen-plasma-bonded to thick PDMS layers with a recipe of 1 min at medium RF level (Plasma cleaner PDC-32G, Harrick plasma). The advantages of doing this were that it was easy to manually align the thick layers while PDMS was transparent and comfortably connected the tubing to deliver liquid into the Flexdym device during the experiment. To manually bond two layers, first both surfaces of the Flexdym channels were plasma-treated with the same recipe and then brought under an optical microscope immediately to align the two layers by focusing the microscope on both surfaces and placing one on top of the other in identical locations to bond. Gently pressing together to remove visible interfacial air gaps is recommended for good bonding, followed by placing it in a 90 °C oven for 5 min. No intermediate adhesive layer was introduced.

### 2.3. Surface Roughness and Power Spectral Density Analysis by AFM

The surface morphology and roughness of the convex PDMS and concave Flexdym microchannels were characterized using a Bruker Dimension Icon Atomic Force Microscope (AFM) (Bruker, Santa Barbara, CA, USA) (Materials Characterization Laboratory, University at Buffalo) operated in tapping mode (Supplementary Figures S4 and S5). A TESPA-V2 silicon probe with a nominal tip radius of ~7 nm, resonance frequency of ~320 kHz, and spring constant of ~37 N/m was used to obtain high-resolution topographical images while minimizing deformation or damage to the PDMS surface, showing that Flexdym has good resistance to abrasion.

Prior to imaging, the reflected laser was aligned on the photodetector, and the probe position was optimized using the integrated optical microscope. After engaging the probe with the sample surface, scan parameters were adjusted to obtain stable imaging and good agreement between the trace and retrace profiles. Bow correction was applied when necessary to improve trace–retrace matching. AFM topographical images of both concave and convex structures were acquired over a 10 µm × 10 µm scan area. The acquired height images were processed using NanoScope Analysis software (Bruker, Santa Barbara, CA, USA). Each image was subjected to first-order flattening to remove sample tilt and low-order background variations while preserving the surface topographical features. Surface roughness was subsequently quantified from the flattened images using the average surface roughness (Ra).

To further evaluate the spatial characteristics of the surface morphology, power spectral density (PSD) analysis was performed on the processed AFM height data using the 2D isotropic PSD function in NanoScope Analysis. The isotropic PSD represents the spatial frequency distribution of surface height variations independent of scan direction and was used to compare the characteristic surface texture and spatial roughness features of convex PDMS and concave Flexdym microchannels.

### 2.4. Scanning Electron Microscopy (SEM)

Surface morphology of the PDMS microstructures was examined using a Hitachi S-4000 Field Emission Scanning Electron Microscope (FESEM) (Hitachi Tech Corporation, Tokyo, Japan) equipped with an IXRF energy-dispersive X-ray spectroscopy (EDS) (IXRF, Inc., Austin, TX, USA) system at the South Campus Instrument Center, University at Buffalo, under the guidance of Dr. Peter Bush. Prior to imaging, the PDMS chips were gold sputter-coated for 60 s at the Materials Characterization Laboratory (MCL), University at Buffalo north campus, to reduce surface charging and improve image quality. The coated chips were then mounted on conductive carbon tape, which provided mechanical stability and electrical contact with the grounded sample stub to facilitate charge dissipation during imaging. The resulting SEM images were used to evaluate the surface morphology and structural features of the fabricated microstructures.

## 3. Results and Discussions

### 3.1. Temperature-Dependent Thermomechanical Framework for PDMS-to-Flexdym Replication

Thermal replication of thermoplastic polymer is governed by the combined effects of temperature-dependent material behavior and activation energy for plastic flow response [44]. Heating across the glass transition temperature region sharply reduces shear modulus, accompanied by a sharp decrease in resistance to polymer-chain slippage, increasing the deformability of the Flexdym substrate and facilitating its conformity to the PDMS convex microstructures, and therefore increasing its replication fidelity. Upon cooling, the increased mechanical stiffness stabilizes the replicated geometry. Therefore, the elastic–viscoplastic behavior of the polymer dominates the deformation of the polymer flow and replication [45]. Strain decomposition and stress relaxation can be phenomenologically explained for thermoplastic replication. The total strain is decomposed into elastic and plastic components, $\varepsilon_{t}=\varepsilon_{el}+\varepsilon_{pl}$. The elastic strain is represented by Hooke’s law, $\varepsilon_{el}=\frac{\sigma_{t}}{E}$, where *ε*_t_ is the total strain *ε*_el_ is the elastic strain, *ε*_pl_ is the plastic strain, characterized using the true stress–strain curves obtained in the uniaxial compression tests, *σ*_t_ is the true stress, and *E* is the elastic modulus. A compact equation that explains the sharp behavior of the material’s property across *T*_g_ [44,46]_,_ $X\left(T\right)=\frac{X_{g}+X_{r}}{2}-\frac{X_{g}-X_{r}}{2}tanh\;[\frac{T-T_{g}}{\Delta }]-L_{X}(T-T_{g})$, where *X(T)* denotes a temperature-dependent material parameter, *X*_g_ and *X*_r_ are its glassy- and rubbery-state values, and *L*_X_ is a parameter-specific linear temperature slope when such a correction is included. The time-dependent viscoelastic behavior well above *T*_g_ during microreplication can additionally be described by the stress-relaxation exponential relation [45], $\sigma_{r}\left(t\right)=aexp\left(-bt\right)+g,$ where *σ_r_*(*t*) is the relaxation stress, a and b are fitted material constants, *t* is the relaxation time, and g is the equilibrium stress. Thus, increasing holding time causes *σ_r_*(*t*) to decrease toward the equilibrium value g. Increasing the holding time allows greater stress relaxation in the softened thermoplastic, reducing its resistance to deformation and thereby improving conformity to the convex microchannel shape of the compliant PDMS mold.

### 3.2. Temperature-Dependent Dimensional Fidelity in PDMS-to-Flexdym Replication

**Figure 2:**
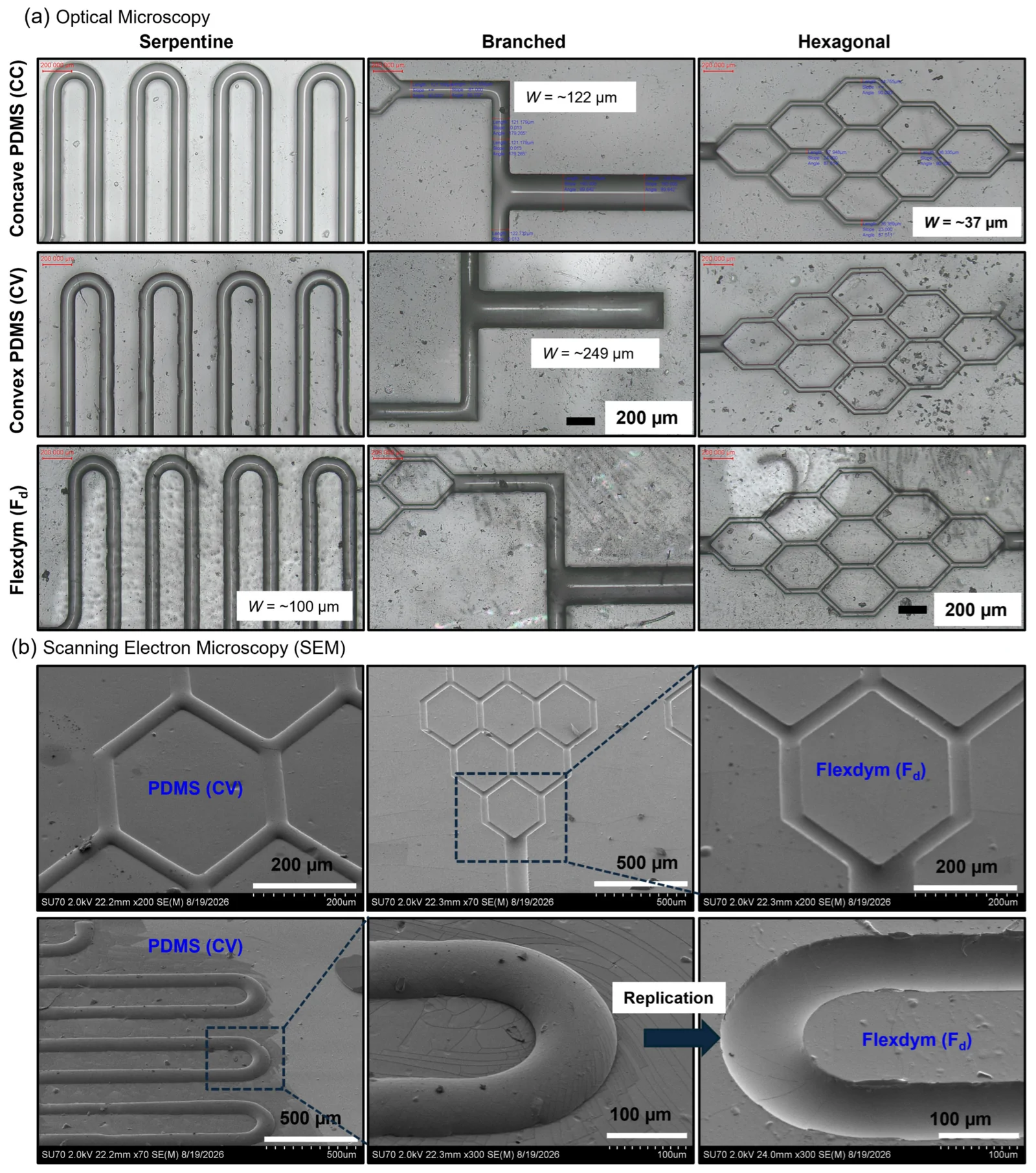
Representative optical microscopy and scanning electron microscopy (SEM) images demonstrating geometric replication of complex microchannel architectures from PDMS to Flexdym. (**a**) Optical micrographs of serpentine, branched, and interconnected hexagonal channel patterns across CC, CV, and F_d_ stages, illustrating preservation of continuous channel paths and overall pattern geometry over representative widths of approximately 37–249 µm. (**b**) SEM images of representative CV and corresponding F_d_ replicas at increasing magnification, highlighting the transfer of hexagonal junctions and smoothly curved serpentine features with well-defined boundaries and local structural fidelity after thermal replication. Scale bars are indicated in each image.

The overall microchannel architectures were well preserved after replication, with serpentine, branched, and hexagonal designs. All retained clear, continuous flow paths and well-defined boundaries, as shown in Figure 2a. SEM imaging further confirmed the local structural fidelity of the replicated Flexdym microchannels, revealing smooth U-turns, distinct junctions, and well-defined sidewalls without major collapse or discontinuity, as shown in Figure 2b. Together, these observations demonstrate reliable PDMS-to-Flexdym replication across both simple and spatially varying complex round microchannel geometries.

**Figure 3:**
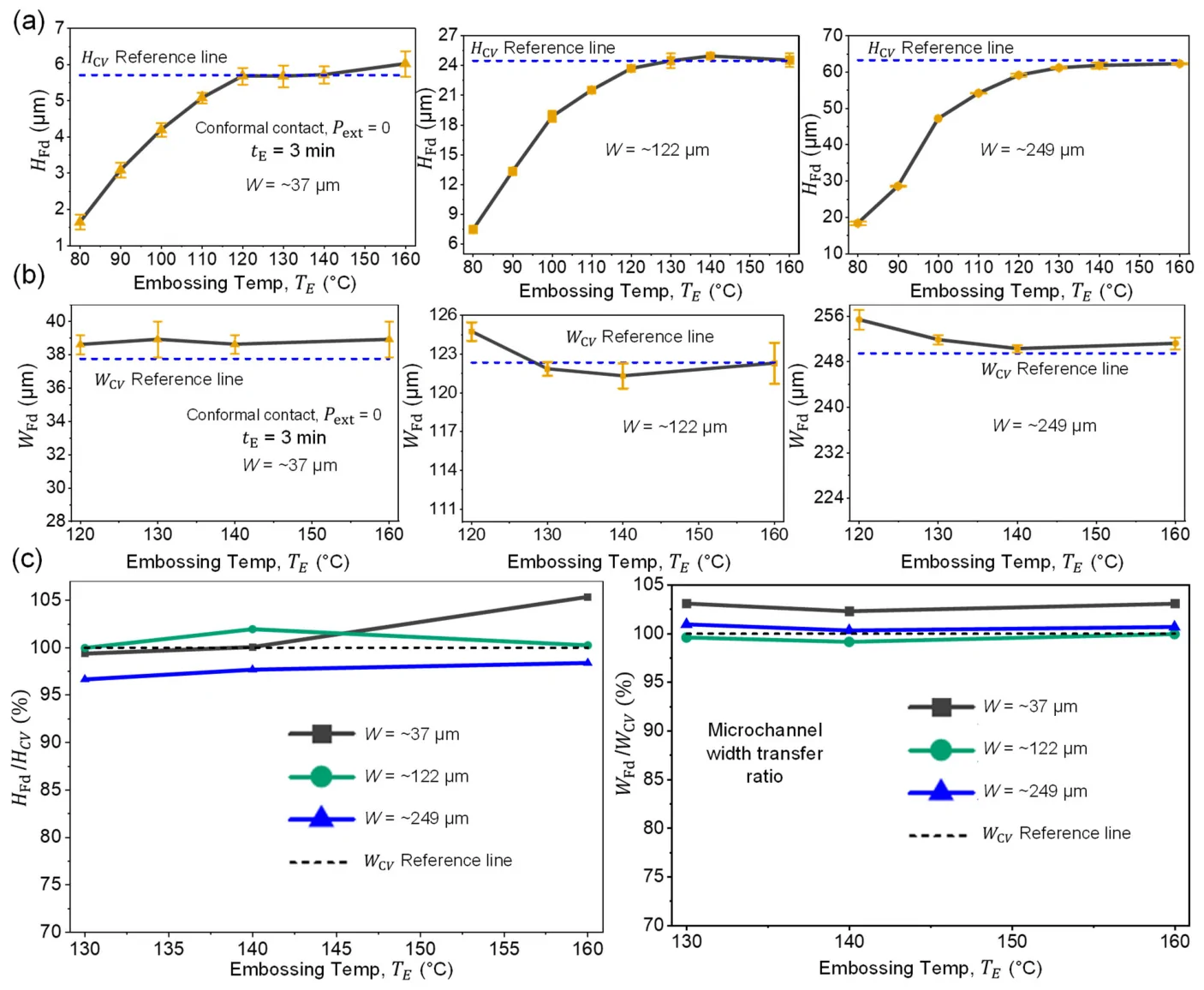
Temperature-dependent dimensional fidelity of PDMS-to-Flexdym thermal replication. (**a**) Replicated *H*_Fd_ as a function of embossing temperature, *T*_E_, for three representative *W* values of ~37 µm, ~122 µm, and ~249 µm, showing progressive recovery of the original channel height with increasing temperature and convergence toward the corresponding *H*_CV_ values. (**b**) Replicated *W*_Fd_ over the same temperature range, demonstrating comparatively limited lateral dimensional variation and close agreement with *W*_CV_. (**c**) Height and width transfer ratios calculated relative to the corresponding convex PDMS molds, showing convergence toward unity at elevated embossing temperatures and identifying the temperature range associated with high-fidelity three-dimensional replication. Data are presented as mean ± SD; dashed lines indicate the corresponding CV values.

To define the thermal process window for direct PDMS-to-Flexdym replication upon conformal contact, no excess external pressure was applied (*P*_ext_ = 0; Supplementary Figure S3), and therefore, the lateral and vertical dimensions of the replicated microchannels were quantified as a function of embossing temperature (*T*_E_) for a fixed embossing time (*t*_E_) of 3 min, as shown in Figure 3a,b.

As representative dimensions across a wide range, three convex PDMS microchannel heights (*H*_CV_) of approximately ~5 µm, ~24 µm, and ~63 µm for the widths (*W*_CV_) of ~37 µm, ~122 µm, and ~249 µm, respectively, were selected for PDMS-to-Flexdym replication and subsequent measurements, as shown in Figure 3a. The replicated Flexdym channel height (*H*_Fd_) showed a significant dependence on *T*_E_. At 80 °C, the replicated *H*_F_ were only 1.65 ± 0.21 µm, 7.47 ± 0.31, and 18.35 ± 0.45 µm, respectively, and the relative difference in height ((*H*_Fd_ *− H*_CV_*)/H*_CV_) = ~70% for all cases. Interestingly, increasing *T*_E_ progressively increased the transferred channel height. At 120 °C, the replicated *H*_F_ relative difference is ((*H*_Fd_ *− H*_CV_*)/H*_CV_) = 6.6% for the *W* = ~249 µm feature, which is already a strong replication quality. By 130 °C, the relative difference goes down to 3.4% for the corresponding *W* = ~249 µm feature. From and above 130 °C, the *H*_F_ response approached a perfect plateau, with reference to the *H*_CV_ as shown in Figure 3a. The increasing *T*_E_ predominantly enhances the thermal softening of Flexdym, therefore increasing *H*_F_. *T*_E_ ≥ 120 °C enables enough softening of Flexdym, allowing the material to conform more completely; therefore, the *H*_F_ reaches very close to the *H*_CV_. As representative dimensions, three convex PDMS microchannel widths (*W*_CV_) of approximately ~37 µm, ~122 µm, and ~249 µm were selected for PDMS-to-Flexdym replication and subsequent measurements, as shown in Figure 3b. The replicated Flexdym channel width (*W*_Fd_) remained close to the corresponding convex *H*_CV_ in the range of *T*_E_ from 120 °C to 160 °C. Between that *T*_E_ interval, the replicated *W*_Fd_ varied only from 38.61 ± 0.58 µm to 38.92 ± 1.08 µm for the ~37 µm feature, from 121.31 ± 0.98 µm to 124.74 ± 0.71 µm for the ~122 µm feature, and from 250.29 ± 0.69 µm to 255.37 ± 1.73 µm for the ~249 µm feature. The corresponding maximum relative differences in width (*W*_Fd_ *− W*_CV_*)/W*_CV_ over that *T*_E_ interval were 2.35%, 1.95%, and 3.08%, respectively, demonstrating that widths were largely preserved across the range of *T*_E_ ≥ 120 °C.

The dimensional fidelity of the replication process is explained by a highly sensitive transfer ratio, as shown in Figure 3c. Width-transfer (*W*_Fd_*/W*_CV_) ratios remained between 99.15% and 103.08% across all three channel sizes and processing temperatures *T*_E_ ≥ 120 °C. The height transfer ratios (*H*_Fd_*/H*_CV_) ranged from 93.41% to 105.35% and 96.64% to 105.35% 9 across all three channel sizes and processing temperatures *T*_E_ ≥ 120 °C and *T*_E_ ≥ 130 °C, respectively. Overall, the convergence of both (*W*_Fd_*/W*_CV_) and (*H*_Fd_*/H*_CV_) toward 100% at *T*_E_ ≥ 130 °C indicates near-complete reproduction of the convex PDMS geometry in Flexdym within this processing range that is recommended; however, *T*_E_ ≥ 120 °C still provides high-quality replication and usable round microchannels for microfluidic applications with only 1 µm to 2 µm difference in (*H*_Fd_*-H*_CV_) or (*W_Fd_-W*_CV_), which is very difficult to achieve even with conventional light-source-based 3D printers having a limited resolution and uncured resin removal constraints for printing <500 µm round cross-sectional channels [47]. Our experimental results have good agreement with the process window of *T*_g_ + 20 ≤ *T*_E_ ≤ *T*_g_ + 40, suggested by previous numerical and experimental work [45]. Increasing temperature above *T*_g_ promotes thermoplastic flow and replication.

### 3.3. Geometric Fidelity Across Sequential PDMS-to-Flexdym Replication Stages

**Figure 4:**
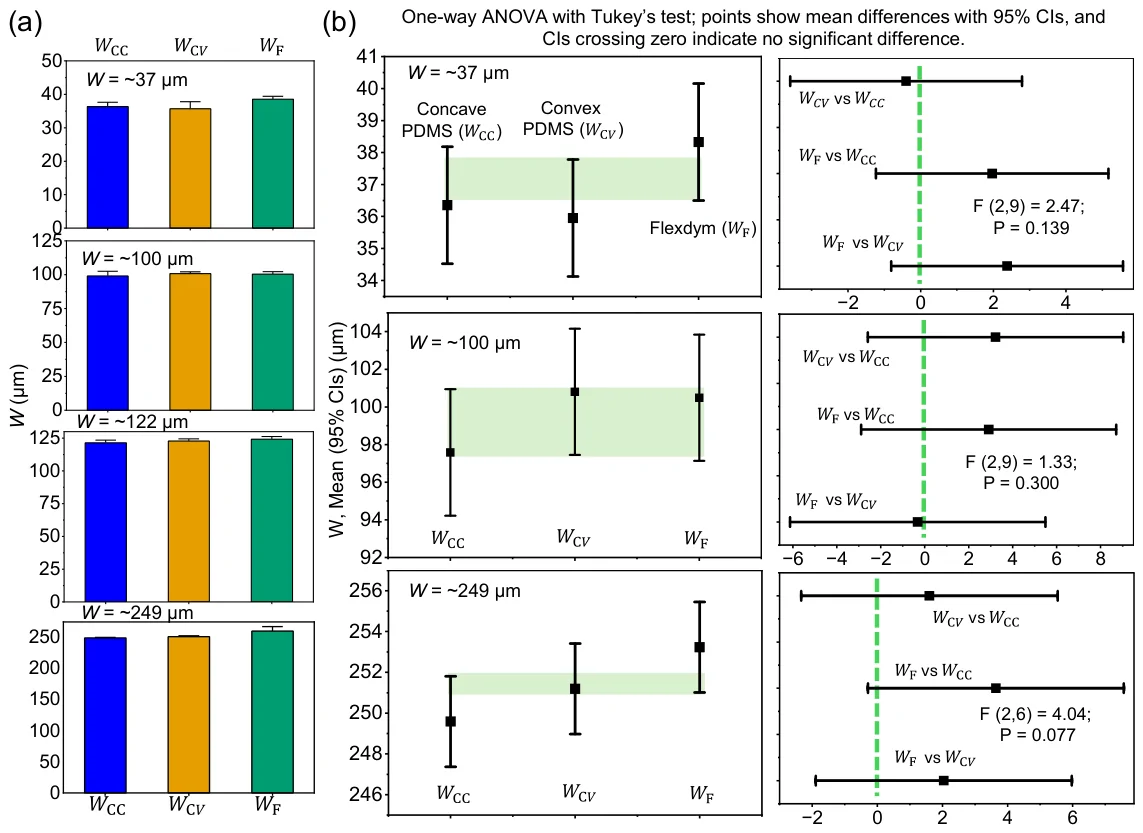
Geometric fidelity of channel width across sequential PDMS-to-Flexdym replication stages. (**a**) Comparison of *W* measured for CC, CV, and replicated F_d_ across four representative *W* of ~37 µm, ~100 µm, ~122 µm and ~249 µm features. Data are presented as mean ± SD. (**b**) Tukey-adjusted pairwise mean differences following one-way ANOVA for the corresponding replication stages for *W* of ~37 µm, ~100 µm, and ~249 µm features; horizontal bars indicate simultaneous 95% confidence intervals, and intervals crossing zero indicate no statistically significant difference. No significant differences in *W* were observed among the three fabrication stages for any feature investigated.

**Figure 5:**
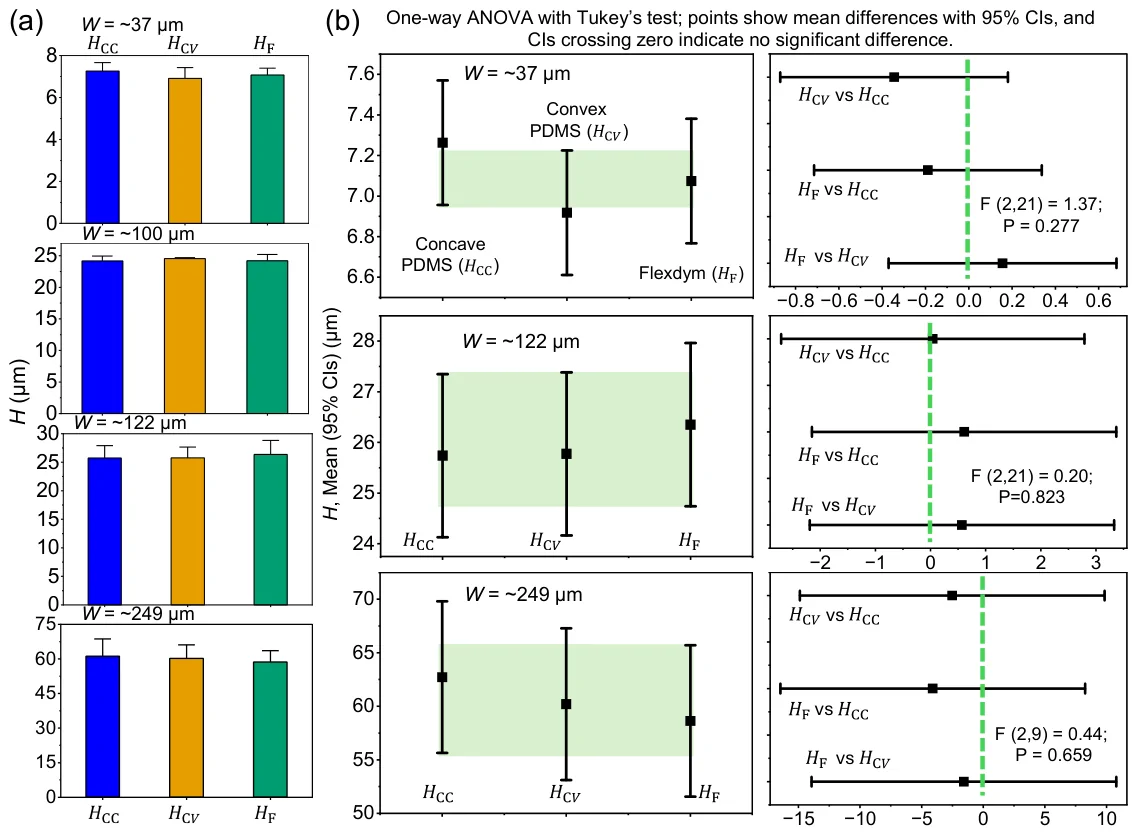
Geometric fidelity of channel height across sequential PDMS-to-Flexdym replication stages. (**a**) Comparison of *H* measured for CC, CV, and replicated F_d_ across four representative *W* of ~37 µm, ~100 µm, ~122 µm and ~249 µm features. Data are presented as mean ± SD. (**b**) Tukey-adjusted pairwise mean differences following one-way ANOVA for the corresponding replication stages for *W* of ~37 µm, ~122 µm, and ~249 µm features; horizontal bars indicate simultaneous 95% confidence intervals, and intervals crossing zero indicate no statistically significant difference. No significant differences in *W* were observed among the three fabrication stages for any feature investigated.

A statistical approach was taken to evaluate geometric fidelity throughout the PDMS-to-Flexdym replication stages. *W* and *H* were compared among the CC, CV, and replicated F_d_ for four representative identical microchannel sizes as shown in Figure 4 and Figure 5. Measurements obtained from multiple locations within the same device were averaged so that each independently fabricated device contributed one value to the analysis. For each nominal channel size, CC, CV, and F_d_ were compared using one-way analysis of variance (ANOVA), followed by Tukey’s post hoc multiple-comparison test. Pairwise results were displayed as Tukey-adjusted mean differences with simultaneous 95% confidence intervals (CIs); CIs excluding zero were considered statistically significant. *W* and *H* measurements are presented as mean ± standard deviation (SD) (Supplementary Table S1A,B). Statistical significance was defined as *p* < 0.05. All statistical analyses were performed using Minitab StatisticalSoftware 22 and plotted in OriginPro 2026 (University at Buffalo license). The measured *W* remained closely preserved throughout the replication steps, as shown in Figure 4a.

For the ~37 µm, ~100 µm, ~122 µm, and ~249 µm features, the replicated *W_Fd_* values were 38.326 ± 0.779 µm, 100.489 ± 1.797 µm, 124.830 ± 2.240 µm, and 253.230 ± 2.020 µm, respectively, compared with corresponding *W*_CV_ of 35.950 ± 2.360 µm, 100.805 ± 1.315 µm, 122.868 ± 1.512 µm, and 251.191 ± 1.590 µm. Relative to *W*_CV_, *W_Fd_* changed approximately by ((*W_Fd_* − *W*_CV_*)/W*_CV_) ≈ +6.61%, −0.31%, +1.60%, and +0.81%, respectively, indicating limited lateral dimensional variation during the final thermoplastic replication step (*n* ≥ 3). Consistent dimensional measurements and one-way ANOVA analysis showed no statistically significant differences among the three stages, with *p* = 0.139, 0.300, 0.187, and 0.077 for *W* of ~37 µm, ~100 µm, ~122 µm, and ~249 µm channels, respectively. Tukey post hoc comparisons were also nonsignificant for all pairwise comparisons, as shown in Figure 4b; the closest comparison was between CC and F_d_ for the ~258 µm feature (*p* = 0.139), with only ~3.64 µm difference. Overall, these results indicate that sequential PDMS inversion and Flexdym replication preserved the lateral channel dimensions across wide ranges.

After measurements and statistical analysis for *H* across ~37 µm, ~100 µm, ~122 µm, and ~249 µm features, *H*_Fd_ were 7.074 ± 0.328 µm, 24.230 ± 0.966 µm, 26.350 ± 2.470 µm, and 58.625 ± 4.955 µm, respectively, compared with corresponding *H*_CV_ of 6.918 ± 0.504 µm, 24.573 ± 0.123 µm, 25.775 ± 1.887 µm, and 60.200 ± 5.897 µm as shown in Figure 5a. Relative to *H*_CV_, *H*_Fd_ changed ((*H*_Fd_ *− H*_CV_*)/H*_CV_) approximately by +2.25%, −1.40%, +2.23%, and −2.61%, respectively, indicating minimal variation in *H* during the final thermoplastic replication step (*n* ≥ 4). In addition, one-way ANOVA showed no statistically significant differences among the CC, CV, and replicated F_d_ stages for any feature size, with *p* = 0.277, 0.724, 0.823, and 0.659 for the ~37 µm, ~100 µm, ~122 µm, and ~249 µm channels, respectively, as shown in Figure 5b. Tukey post hoc comparisons were likewise nonsignificant for all pairwise comparisons as before in *W*. Overall, these results demonstrate that the sequential PDMS inversion and Flexdym replication steps produced only minimal variation in *H*, supporting preservation of the vertical dimensions across a broad range of channel sizes. The preservation throughout the dimensions is particularly important for retaining the intended rounded cross-sectional geometry, because changes in the vertical dimension would directly alter the fluidic resistance and hence, fluidic performance of the device. For laminar flow, the hydraulic resistance, *R*_H_, is inversely proportional to the fourth power of the hydraulic radius (*R*_H_ ∝ *r*_H_^−4^), indicating that even small reductions in *r*_H_ essentially increase flow resistance [48]. The minimal variation was observed across all four feature sizes; therefore, our thermal replication method supports reliable three-dimensional transfer of the original PDMS microchannel geometry into a thermoplastic Flexdym substrate.

**Figure 6:**
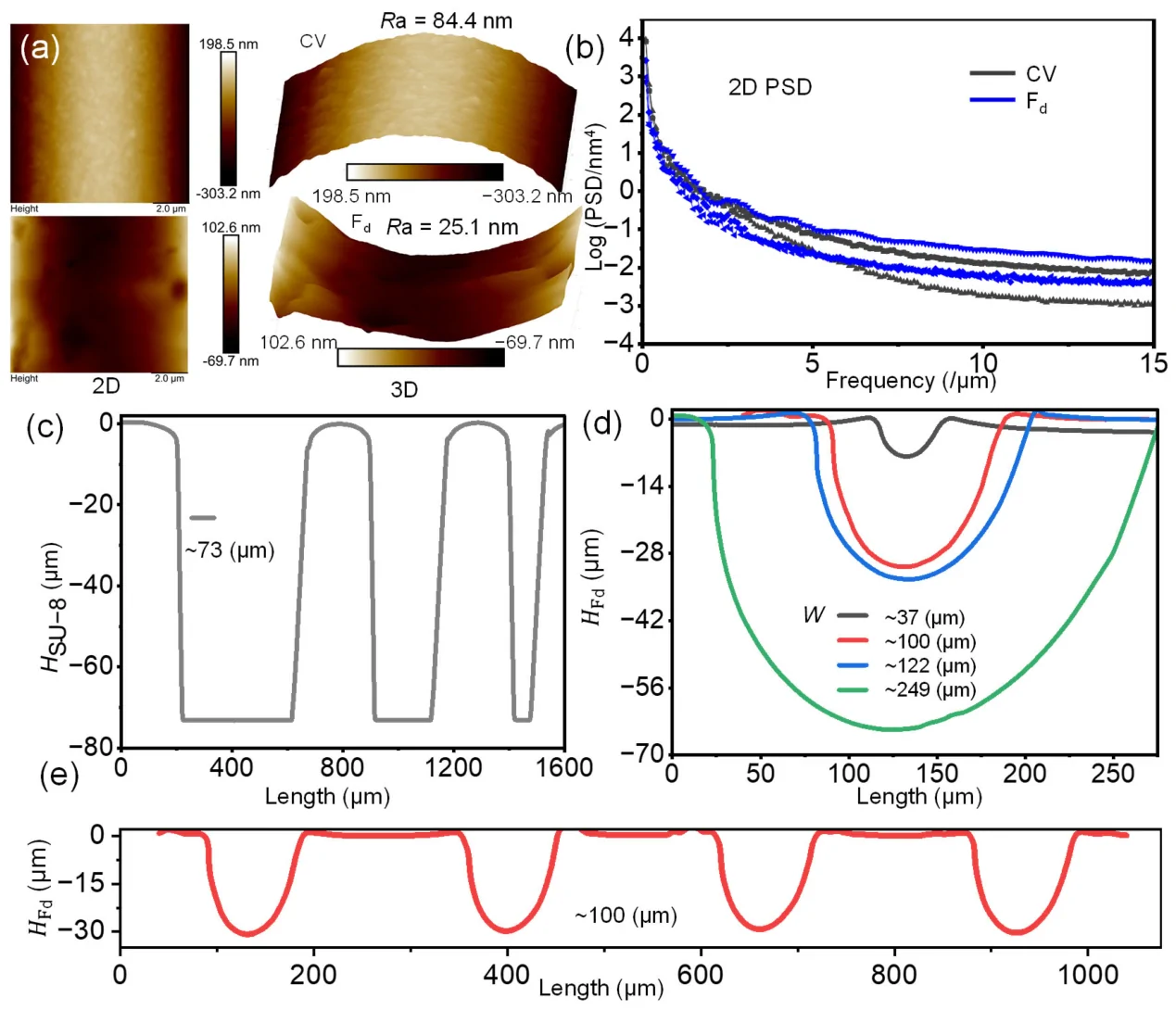
Surface and cross-sectional characterization of replicated Flexdym microchannels. (**a**) AFM topographies of CV (convex PDMS mold) and F_d_ (replicated Flexdym microchannel) surfaces, with average roughness values of approximately 84.1 and 25.1 nm, respectively (Supplementary Figures S4 and S5). (**b**) Corresponding power spectral density profiles. (**c**) Stylus-profilometry trace of the original SU-8/silicon master. (**d**) Overlaid Flexdym cross-sectional profiles demonstrating preservation of rounded channel morphology across different dimensions. (**e**) Repeated profilometry profiles showing consistent channel shape and depth, supporting replication reproducibility.

**Figure 7:**
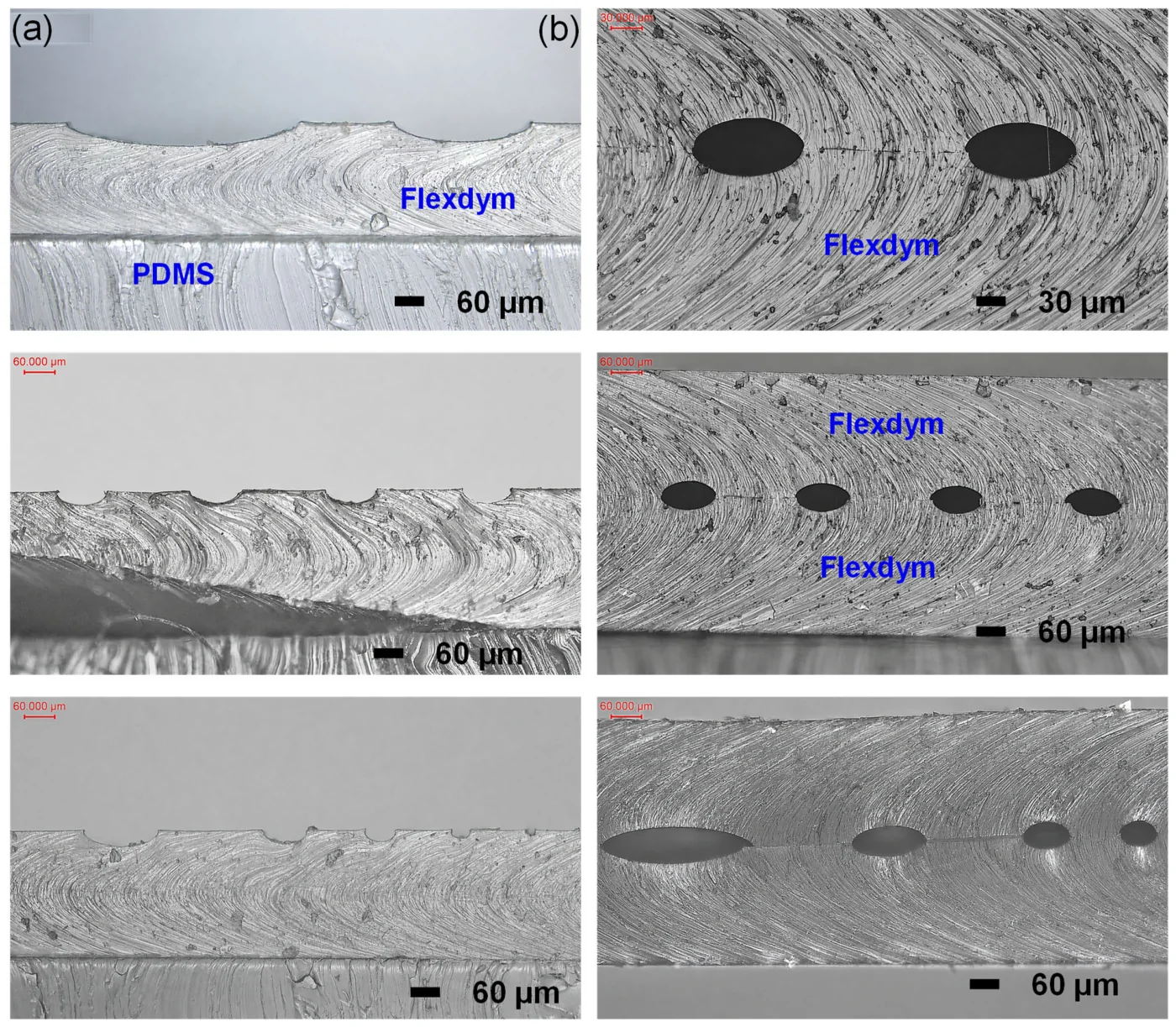
Cross-sectional characterization of aligned semi- and full-round Flexdym microchannels. (**a**) Optical cross-sectional images of replicated semi-round channels formed at the PDMS–Flexdym interface, demonstrating preservation of the curved channel profile. (**b**) Cross-sectional images after alignment and assembly of complementary semi- round channels, showing formation of enclosed round microchannels with continuous interfaces and well-defined geometries. Scale bars are indicated in each image.

AFM characterization further assessed the surface quality of the replicated structures (Supplementary Figures S4 and S5). The 2D and 3D topographic maps showed a reduction in average surface roughness from approximately 84.1 nm for CV to 25.1 nm for *F*_d_, as shown in Figure 6a, indicating that the thermal replication process did not introduce substantial surface degradation. Consistently, the corresponding power spectral density profiles exhibited similar frequency-dependent decay for both surfaces, as shown in Figure 6b, supporting the preservation of the overall surface texture characteristics after PDMS-to-Flexdym thermal replication. Furthermore, stylus profilometry of the original SU-8/silicon master revealed a well-defined negative microchannel template with uniform feature height (*H*_SU-8_), prior to round microchannel formation, as shown in Figure 6c. Overlaying the cross-sectional profiles of the replicated Flexdym channels further demonstrated preservation of the rounded morphology across the investigated dimensions, with channel widths ranging from 38.56 ± 0.76 µm to 260.31 ± 6.14 µm and corresponding heights from 7.04 ± 0.31 µm to 58.63 ± 4.29 µm, as shown in Figure 6d. As demonstrated in our previous work, the *H*_CC_ can be systematically tuned through pre-curing temperature, pre-curing time, channel width, and initial trapped-air volume [16,42]. All profiles preserved smooth curved sidewalls and rounded bottoms, indicating successful transfer of the intended round geometry, as shown in Figure 6e. Cross-sectional optical micrographs of the fabricated Flexdym microchannels before and after alignment are shown in Figure 7. The semi-circular microchannel profiles, as shown in Figure 7a, demonstrate preservation of the curved geometry across different channel dimensions as well as the repeatability of the same *W* of ~100 µm microchannels. Following microscopic manual alignment of complementary semi-round channels, the formation of fully enclosed round microchannels with continuous boundaries without channel collapse is shown in Figure 7b, confirming successful assembly of the replicated microchannels, also validated by SEM results in Figure 2b.

**Figure 8:**
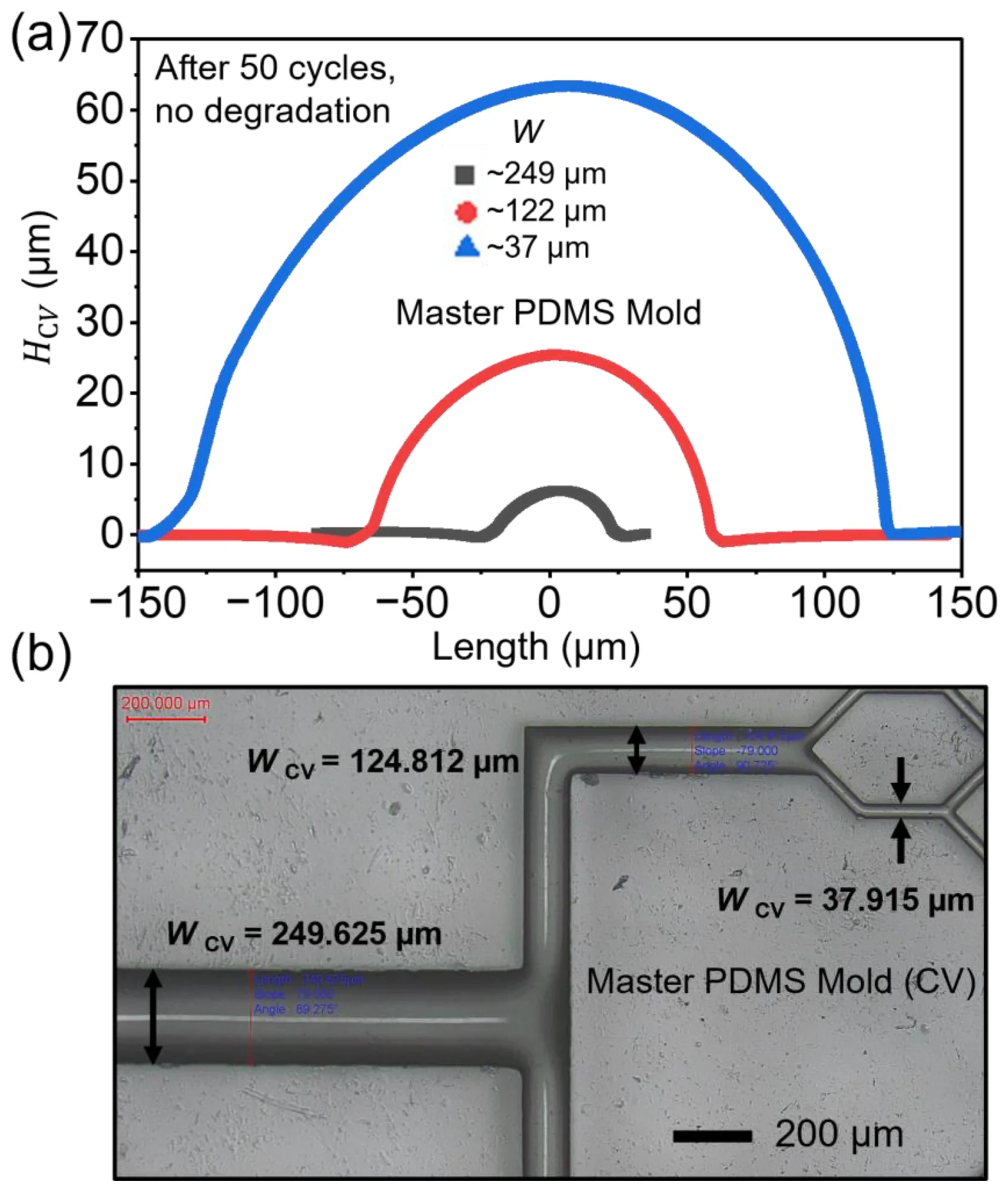
Reusability of compliant PDMS convex mold after 50th cycle of thermal replication process. (**a**) Channel height measured in the PDMS convex mold by profilometry, and (**b**) channel width measured in the PDMS convex mold by optical microscope, showing dimensions are in the expected range as measured previously, with no degradation. Scale bar is indicated in the image (Supplementary Figure S6).

We have evaluated the same convex PDMS mold after 50 consecutive Flexdym thermal replication and demolding cycles under the optimized processing conditions. After the 50th cycle, we did not observe changes in *H*cv, as shown in Figure 8a. Optical microscopy showed corresponding feature widths of 37.915 µm, 124.812 µm, and 249.625 µm, as shown in Figure 8b (Supplementary Figure S6), with no visible cracking, tearing, collapse, attaching, bending, or delamination of the PDMS structures. Therefore, the method looks promising for reusing the compliant PDMS mold again and again once fabricated that will minimize the burden of the fabrication process and make the whole process rapid. As we did not experience any continuous decay in mold quality or dimensional degradation for at least 50 cycles under optimized conditions tested, the method provides good reusability of the compliant PDMS mold.

### 3.4. Two-Phase Syringe-Assisted Compressed Air Microvalve Enabled by Round-Microchannel Integration

**Figure 9:**
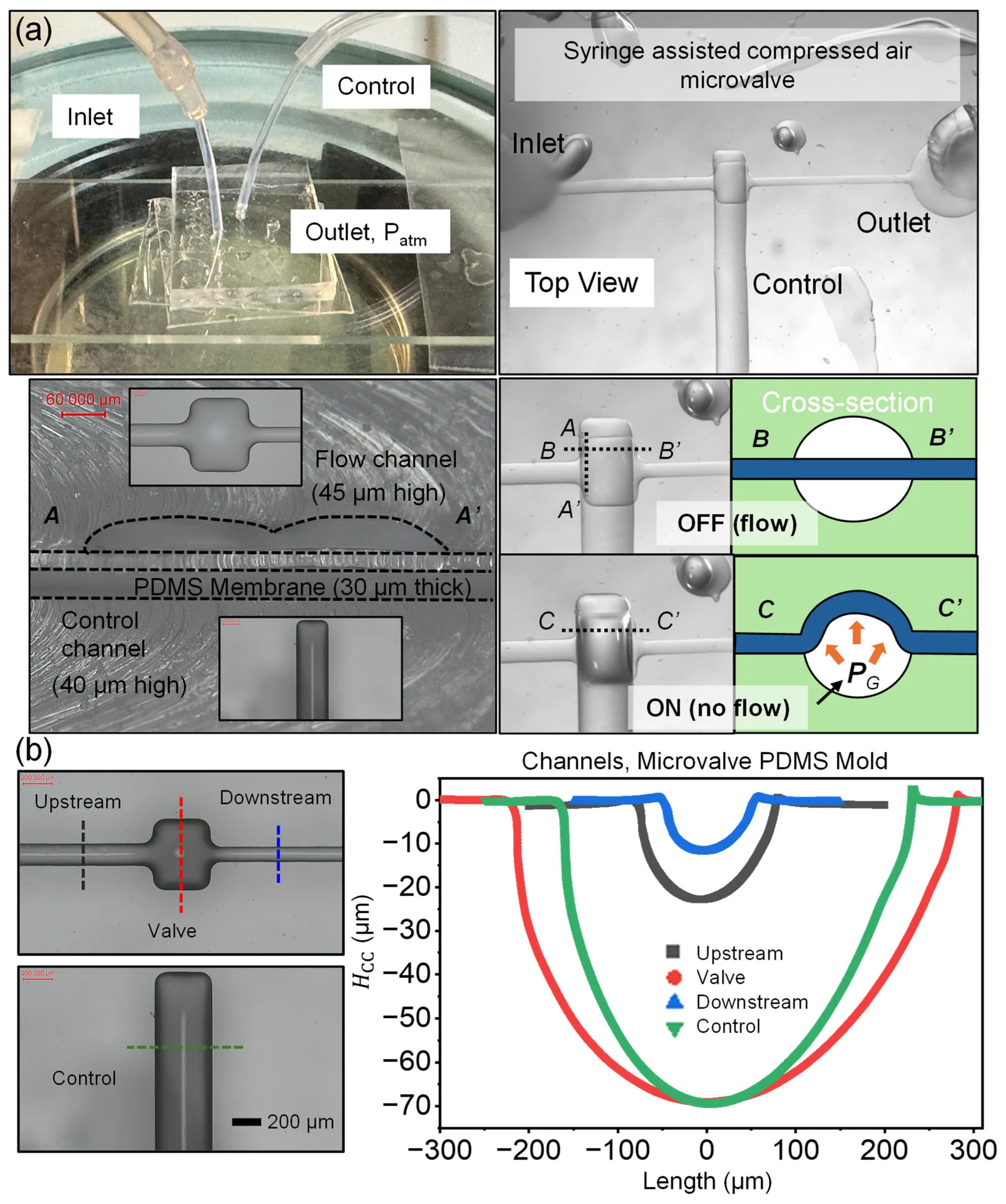
Round-channel-based syringe-assisted compressed air microvalve operation and trapped-air pressurization. (**a**) Schematic and microscopy images of the multilayer microvalve consisting of orthogonally arranged flow and control channels separated by a thin PDMS membrane fabricated by thermal air expansion, PDMS double casting, and thermal replication (Supplementary Figures S7 and S8), demonstrating the OFF (flow) and ON (no-flow) states induced by trapped-air compression (Supplementary Figure S9). A…A’, B…B’ and C…C’ are indicated on the microvalve to show cross sectional profile after cutting, and also OFF, ON state of microvalve during cycle of operation. (**b**) Channel dimensions at different locations (upstream channel, valve, downstream channel and control channel) of the microvalve, measured from the microvalve PDMS concave mold by profilometry replication (Supplementary Figure S7). Scale bar is indicated in the image.

As microfluidic systems become increasingly complex, microvalves are essential for precise and programmable control of fluids within integrated microscale networks [49]. To demonstrate the functionality of fabricated round microchannels in a Flexdym thermoplastic substrate along with a multilayer microfluidic architecture, a two-phase syringe-assisted compressed-air microvalve was implemented using an orthogonally arranged flow and control channel separated by a thin PDMS membrane, as shown in Figure 9a (Supplementary Figures S7 and S8). The PDMS–Flexdym–glass multilayers were aligned and oxygen plasma bonded with the same protocol stated earlier. Monolithic and hybrid bonding of the Flexdym substrate and their bonding strength and pressure resistance have been quantitatively characterized in previous microfluidic studies [3,34,35,36]. These results open the way for enhanced bonding outcomes, covering pressure ranges commonly used in most microfluidic experiments, particularly in life science applications. Therefore, we did not feel the urge and importance of quantifying the bonding strengths, pressure resistance or leakage testing in our devices. The device consisted of a ~23 µm high upstream flow channel, a ~12 µm high downstream flow channel, ~69 µm high both valve and round control channels, and an approximately 30 µm thick middle membrane. The PDMS membrane was fabricated by spin coating on a HMDS coated glass slide and then curing at 110 °C for 20 min on a hotplate. Thereafter, the bottom Flexdym layer with membrane, and then, top Flexdym layer were bonded together via oxygen plasma treatment. To investigate the multilayered cross-sectional view, we cut the device orthogonally with scissors in the position A…A’, and the 2D view at location B…B’ is also understandable. In that precise location, the top flow channel is ~45 µm high, with a ~40 µm high bottom control channel in the valve area. Under the initial OFF (flow) condition, the membrane remained undeformed, and the upper flow channel remained open with an inlet water head pressure of 14.5 kPa. The membrane deflected into the upper channel and produced the ON (no-flow) state while compressing air inside the control channel with a syringe (1.6-inch, 1 mm diameter tube connected to control channel and 10 mL syringe (no water) (Supplementary Figure S9); total calculated initial air volume for experiment *V*_i_ = *V*_Syringe,I_ + *V*_tube_,_I_ = 3 mL + π × (1/2)^2 × 40.64 = 3.0319 mL, compressed to *V*_2_ = 1.0319 mL), assuming isothermal compression (Boyle’s law)). The calculated air pressure will be *P*_G_ = (P_atm_*V*_i_/*V*_2_)-P_atm_ = 196.46 kPa, demonstrating effective flow shutoff without a traditional complex and high-pressure air compression system, and that can potentially be programmable in the future with the reciprocating motion of the stepper motor. The *V*_i_ is consistent throughout the cycle of operation of the microvalve. We neglected the initial air volume of the control channel compared to the large syringe and tube volume to simplify experimental analysis and the analytical model. A syringe with an initial air volume without water is recommended for future programmable design, as we demonstrated here in the experiment, because water is incompressible. However, to simplify the numerical and analytical model, we considered initial water with inflow of water with a constant volumetric flow rate for air compression in the control channel.

The upper round microchannel and its continuously curved cross-section, as shown in Figure 9b, facilitate a more secure fitting of the deflected membrane against the surface than a sharp-edged rectangular microchannel. Therefore, due to no corner leakage, it can provide complete closure [50]. In addition, the use of a round control microchannel may promote more uniform membrane deflection because the compressed air exerts an isotropic pressure that acts locally normal to the continuously curved channel surface, generating a more symmetric distributed load across the membrane edges to the center and facilitating complete valve closure.

#### Analytical Modeling of Syringe-Assisted Air Compression in Microchannels

**Figure 10:**
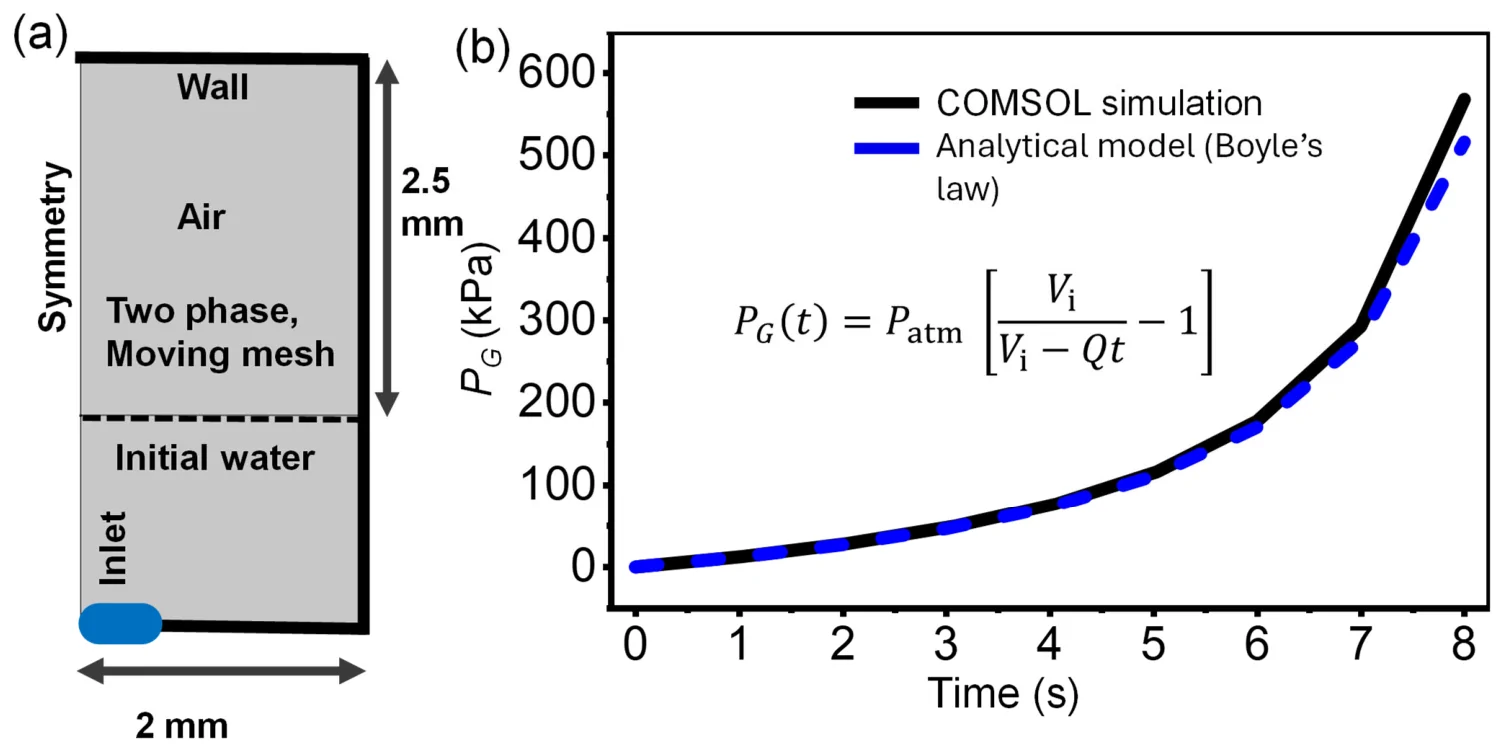
COMSOL simulation of trapped-air compression during water inflow. (**a**) Schematic of the two-phase air–water computational domain and boundary conditions, showing the initial water region, trapped air, inlet, walls, and moving-mesh interface. (**b**) Two-phase air–water COMSOL simulation using a moving-mesh formulation, illustrating continuous decrease in trapped-air volume and corresponding nonlinear pressure increase during constant water inflow. The simulated pressure response agrees with the analytical compression model.

To provide an independent physics-based correlation with the transient COMSOL Multiphysics 6.0 results, trapped air pressurization was described using conservation of liquid volume coupled with isothermal ideal gas compression. Water was treated as incompressible and introduced into the sealed gas-filled domain at a constant volumetric flow rate, Q. For constant water inflow, $\frac{{dv}_{w}}{dt}=Q$. Neglecting leakage, gas dissolution, and mass transfer across the air–water interface, the injected liquid volume after time *t*, is $V_{w}\left(t\right)=Qt.$ The corresponding compressed air volume therefore reduces according to $V_{G}\left(t\right)=V_{i}-Qt,$ where *V*_i_ is the initial air volume. In the numerical model as shown in Figure 10a, *V*_i_ corresponds to the initial air-domain volume. For experimental implementation, *V*_i_ includes the initial syringe and tube volume connected to the control channel. Assuming isothermal air compression inside the microchannel, the trapped air follows Boyle’s law, $P_{G,abs}\left(t\right)V_{f}\left(t\right)=P_{0,abs}V_{i},$ where P_O,abs_ is the initial air pressure that is atmospheric pressure, P_atm_ and *P_G_*_,abs_(*t*) are the instantaneous absolute gas pressure after time *t,* $P_{G,abs}\left(t\right)=P_{0,abs}\frac{V_{i}}{V_{i}-Qt}$. The corresponding gauge pressure of air $P_{G}\left(t\right)=P_{atm}\;[\frac{V_{i}}{V_{i}-Qt}-1]$. A characteristic compression time can be defined as $t_{c}=\frac{V_{i}}{Q},$ which gives the equivalent compact expression $P_{G}\left(t\right)=P_{atm}\frac{t}{t_{c}-t},\;0\leq t<t_{c}.$ This analytical relation predicts an accelerating increase in air pressure as the finite trapped air volume decreases, consistent with the nonlinear response obtained from the two-phase moving-mesh COMSOL simulation as shown in Figure 10b (Supplementary Information). Assuming incompressible water, negligible leakage and interfacial mass transfer, and approximately isothermal gas compression, the trapped air volume decreases continuously with injected liquid volume, and the air pressure consequently increases according to Boyle’s law.

The entire computational domain was defined as a deforming domain using the Moving Mesh interface to track displacement of the air–water interface. The domain was discretized using a free triangular mesh with the COMSOL predefined Fine element size for fluid-flow calculations. A time-dependent study was performed over 0-8 s, and the coupled Multiphysics equations were solved using the PARDISO direct solver. Water was introduced through a fully developed laminar-flow inlet at $Q=200\;\mu L\;min^{-1}$. To make an identical setup with the simulation, the analytical model assumes a syringe is initially filled with water that can provide the same flow rate of $Q=200\;\mu L\;min^{-1}$ in the 1.6-inch connected tube and the control channel, assuming connected to a syringe pump; therefore, the initial air volume in the analytical model will be *V*_i_ = ~31.9 µL, *t*_c_ = *V*_i_/Q = ~9.57 s that is experimentally feasible. The analytical pressure was calculated at the same time points as the numerical solution $P_{G}\left(t\right)=P_{atm}\frac{t}{9.57-t}$, and the analytical results showed good agreement with the simulation. The microvalves can be implemented on-chip microscale networks with careful design considerations [51].

## 4. Discussion and Conclusions

In summary, this work establishes a direct PDMS-to-Flexdym thermal replication strategy for the rapid, direct, simple, and low-cost fabrication of round microchannels with high dimensional fidelity and reproducibility. The proposed approach enables reliable transfer of diverse complex microchannel architectures while maintaining smooth curved cross-sectional profiles, consistent geometrical features, and structural integrity throughout the replication process. The maximum aspect ratios were achieved using the paired mean width (*W*_Fd_) and height (*H*_Fd_) of the replicated semi-round Flexdym channel. Therefore, the maximum experimentally demonstrated height-to-width (*H*_Fd_/*W*_Fd_) ratio was 0.242, corresponding to a width of 100 µm and a height of 24.23 µm. The largest width-to-height ratio was 6.51, obtained for the 37 µm wide and 5.68 µm deep feature. The ratio can be tuned by playing with the parameters of pre-curing time, temperature, and the initial volume of air that is governed by the height of the fabricated SU-8 structures (*H*_SU-8_) during the concave PDMS mold (CC) fabrication by thermal air expansion [16,42]. 

The method is a form of thermal replication/soft embossing distinguished by the use of a compliant, rounded PDMS positive mold and a simple benchtop mechanical contact system. Conventional hot embossing provides accurate replication but generally requires controlled heating, pressure, cooling, and dedicated tooling. Injection molding offers rapid, reproducible, high-volume production but requires expensive molds and equipment. Laser ablation enables rapid, maskless design modification; however, it can produce poor surface finishes, Gaussian-shaped profiles, and thermally induced bulges. PDMS soft lithography is inexpensive and well suited to prototyping, although rounded channels generally require an additional mold-reshaping step, limited to PDMS material only. UV-curable replication provides high-resolution patterning but is restricted by polymer selection, UV-exposure conditions, and the requirement for suitable molds or photomasks [22]. By integrating the design flexibility of PDMS-based rapid prototyping with soft lithography and manufacturing advantages of thermoplastic materials, this strategy provides a viable pathway for translating spatially varying complex round-channel architectures into robust thermoplastic microfluidic platforms. The principal advantages of our method are its relatively minimal and inexpensive equipment and tooling requirements, rapid replication, direct transfer of rounded profiles into Flexdym thermoplastic substrate, conformal contact provided by the compliant reusable PDMS mold, and suitability for iterative prototyping, potentially bridging conventional PDMS soft lithography to direct, rapid, inexpensive and scalable thermoplastic microfluidic manufacturing. Its current limitations include potential thermomechanical deformation of the thin compliant PDMS mold at high temperature (recommended mold thickness 3mm that we used in this work), manual alignment of two semi-circular channel layers to form a complete round microchannel, and a tested dimensional range that does not establish the ultimate resolution or aspect-ratio limit. Accordingly, we position this method as a rapid, direct and low-cost prototyping approach with potential for future scale-up; however, its suitability for high-volume manufacturing requires further validation of automated loading, contact and alignment, process reproducibility, and mold service life.

Future investigations will explore the application of Flexdym-based round microchannels in organ-on-a-chip systems, including endothelial cell culture under controlled flow conditions to investigate vascular mechanobiological responses. Comparative studies between thermoplastic round-channel platforms and conventional PDMS rectangular-channel devices under equivalent hydrodynamic conditions will further elucidate the respective contributions of material compatibility and channel geometry to cellular behavior, while exploiting the advantages of Flexdym in fabrication reproducibility, reduced molecular adsorption, and potential for large-scale manufacturing. The fabricated round-channel platform was successfully integrated into a microvalve architecture as a proof-of-concept, demonstrating the feasibility of employing replicated round microchannels for microscale fluid control.

## Data Availability

The original contributions presented in this study are included in the article/Supplementary Material. Further inquiries can be directed to the corresponding author.

## Supplementary Materials

The following supporting information can be downloaded at: https://www.mdpi.com/article/10.3390/polym18192387/s1.
