# Supplementary material for: Rapid Fabrication of Round Flexdym Microchannels via Direct PDMS (Thermoset)-to-Flexdym (Thermoplastic) Thermal Replication
Source: Polymers (Basel). 2026 Sep 30;18(19):2387. doi: 10.3390/polym18192387 (PMC13667228; doi:10.3390/polym18192387)
Supplement: Supplementary file 1 [file polymers-18-02387-s001.zip › polymers-4567580-supplementary.pdf]

Rapid Fabrication of Round Flexdym Microchannels via Direct PDMS (thermoset)-to-Flexdym (thermoplastic) Thermal Replication

Md Emamul Kabir[1], Sonali Garg[3], Aldrian Muhaxheri[1], Paras N. Prasad[3,4], Kwang W. Oh[1,2*]

[1]SMALL (Sensors and MicroActuators Learning Lab), Department of Electrical Engineering, University at Buffalo, The State University of New York at Buffalo (SUNY-Buffalo), New York, 14260, USA

[2]Department of Biomedical Engineering, University at Buffalo, SUNY, New York, New York 14260, United States

[3]Department of Chemistry, University at Buffalo, SUNY, New York, New York 14260, United States

[4]The Institute for Lasers, Photonics, and Biophotonics, University at Buffalo, SUNY, New York, New York 14260, United States

[*]Correspondence: kwangoh@buffalo.edu

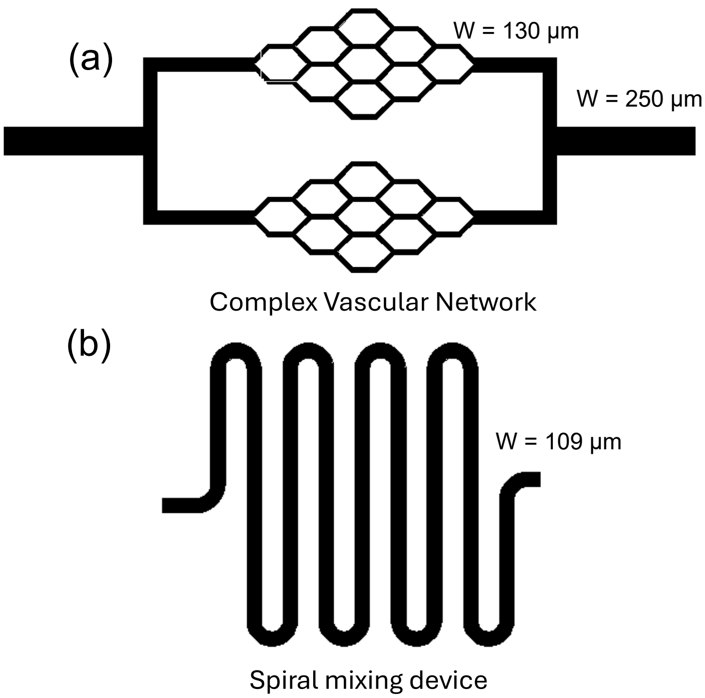

Figure S1. Schematic representation of mask design of microchannel architectures used for replication and characterization. (a) The upper schematic illustrates interconnected branched and hexagonal channel networks with different designed channel widths ($W \approx$ 130 and 250 μm), (b)while the lower schematic shows a serpentine microchannel design channel width ($W \approx$ 109 μm).

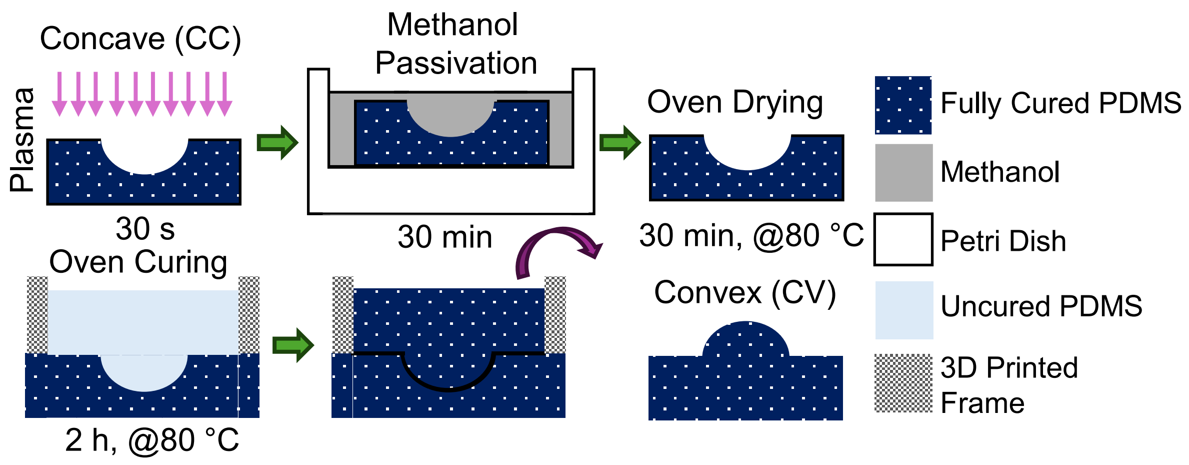

Figure S2. Convex compliant PDMS mold fabrication through PDMS double casting process. The cross sectional view of microchannel structure is shown throughout the process.

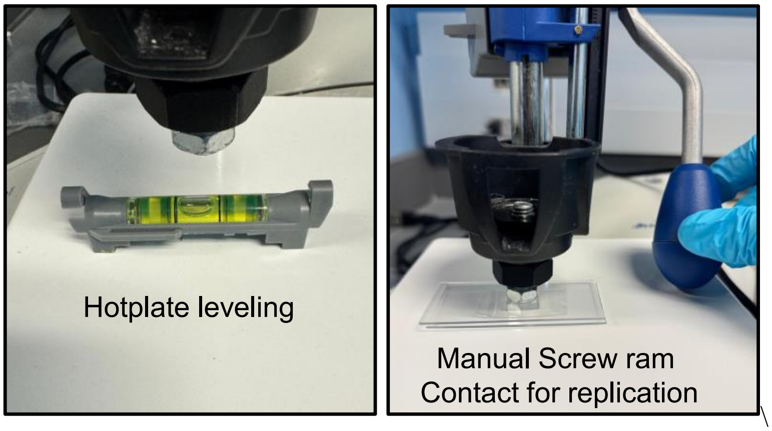

Figure S3. Experimental setup for PDMS-to-Flexdym thermal replication. Photographs showing the replication setup, including (left) hotplate leveling to ensure uniform mold alignment and thermal contact, and (right) manual screw-ram-assisted conformal contact between the PDMS mold and Flexdym substrate during the replication process.

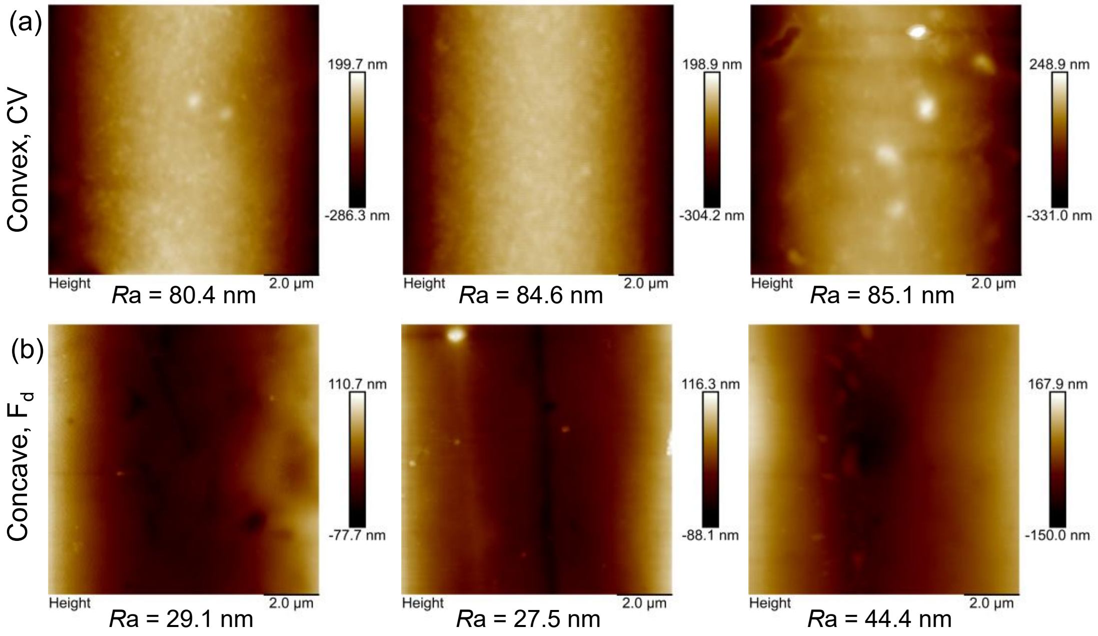

Figure S4. AFM characterization of PDMS molds and replicated Flexdym surfaces. Representative two-dimensional AFM height maps of convex PDMS molds (top row) and corresponding replicated Flexdym surfaces (bottom row) with measured average roughness values (Ra) ranging from 80.4-85.1 nm for PDMS and 27.5-44.4 nm for Flexdym. The reduced surface roughness observed after replication indicates smooth transfer of the mold surface characteristics and demonstrates the suitability of the thermal replication process for producing high-quality thermoplastic microfluidic structures.

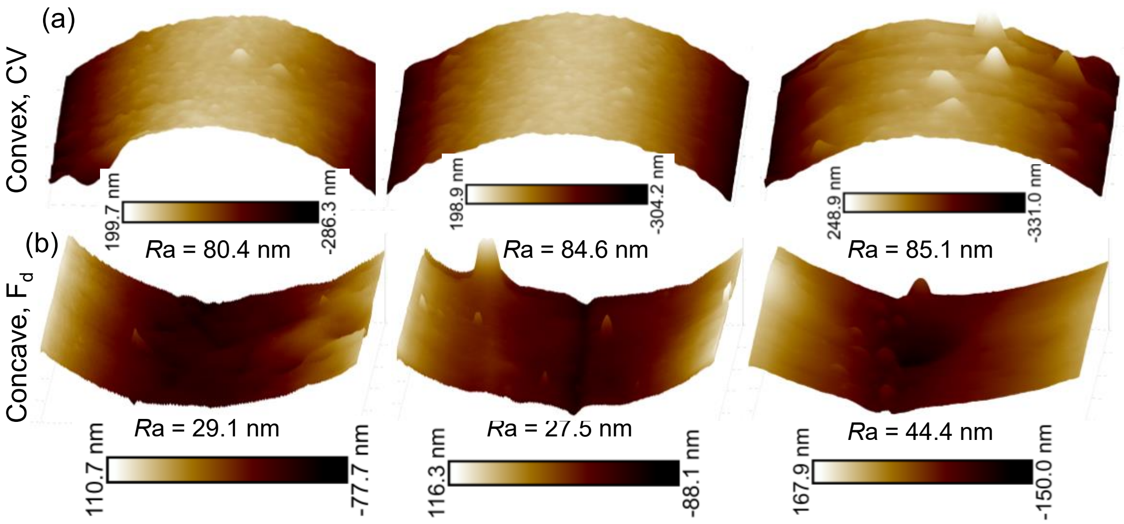

Figure S5. Three-dimensional AFM surface characterization of convex PDMS molds and replicated Flexdym surfaces. Representative 3D AFM height profiles of convex PDMS molds (top row) and corresponding Flexdym replicas (bottom row) showing the preservation of surface morphology after thermal replication. The measured average roughness values (Ra) ranged from 80.4-85.1 nm for PDMS and

27.5-44.4 nm for Flexdym, demonstrating successful transfer of the curved mold features while maintaining smooth replicated thermoplastic surfaces.

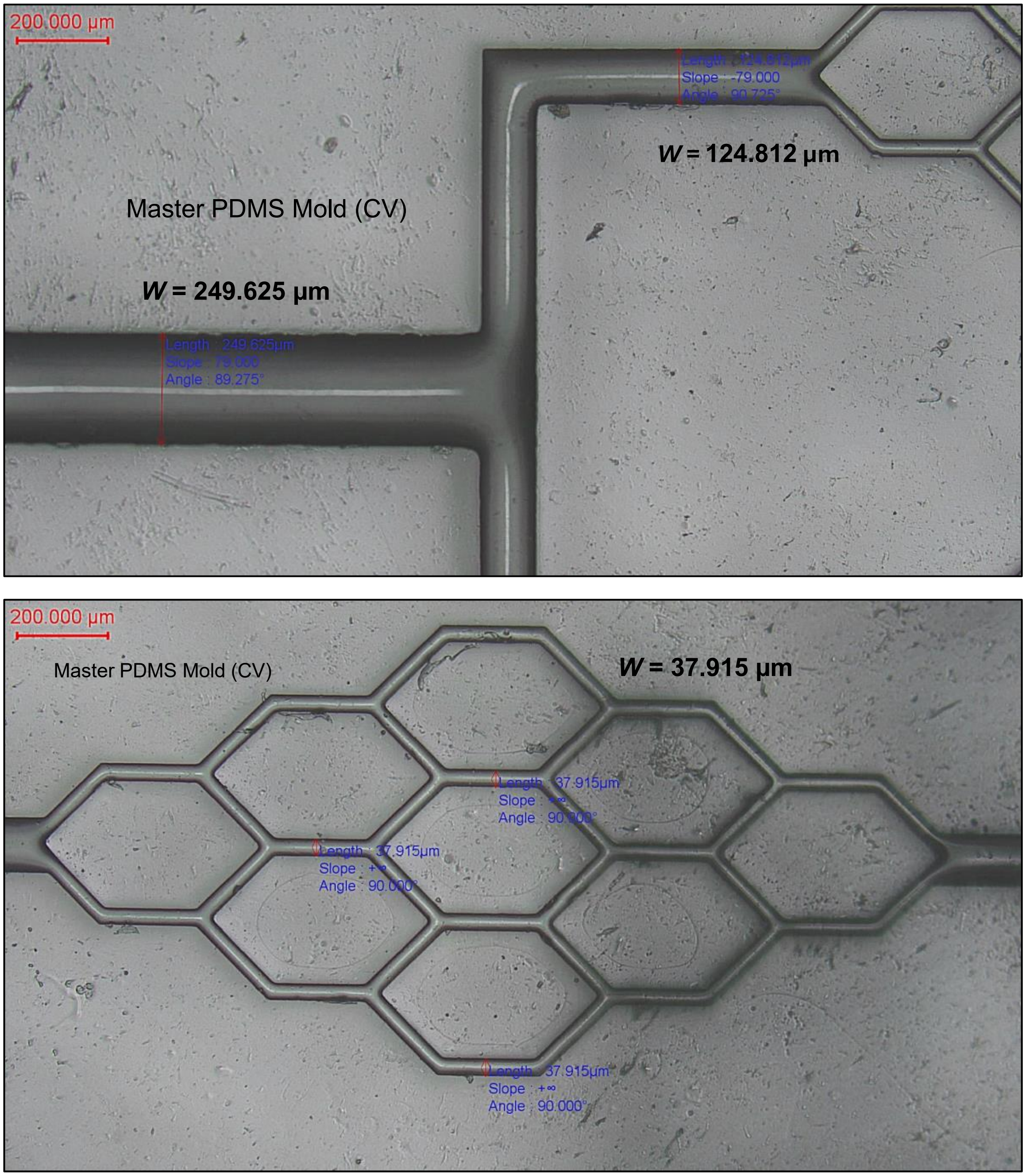

Figure S6. Reusability of compliant PDMS convex mold after 50th cycle of thermal replication process. Channels width measured in the PDMS convex mold by optical microscope, showing dimensions are in the expected range as measured previously, no degradation. Scale bars are indicated in the image.

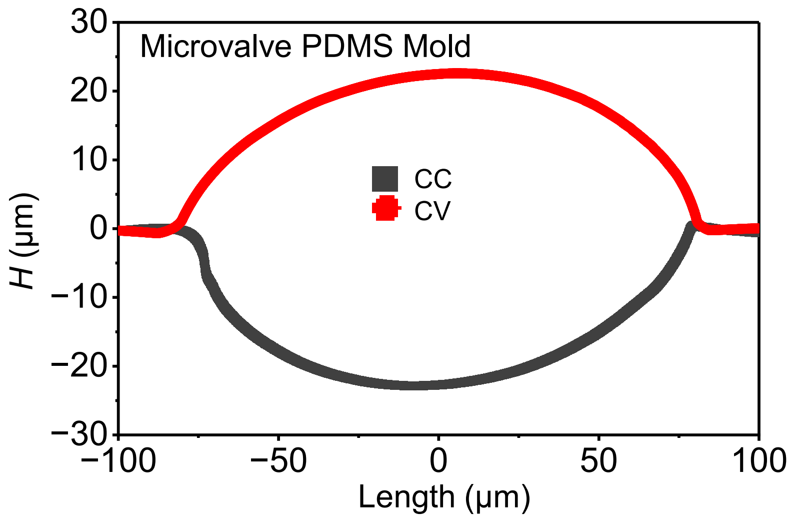

Figure S7. Channel height measured in the microvalve PDMS concave and convex mold by profilometry. Height of the concave and convex channels are similar, indicating reliable PDMS double casting process before thermal replication beginning of the microvalve fabrication.

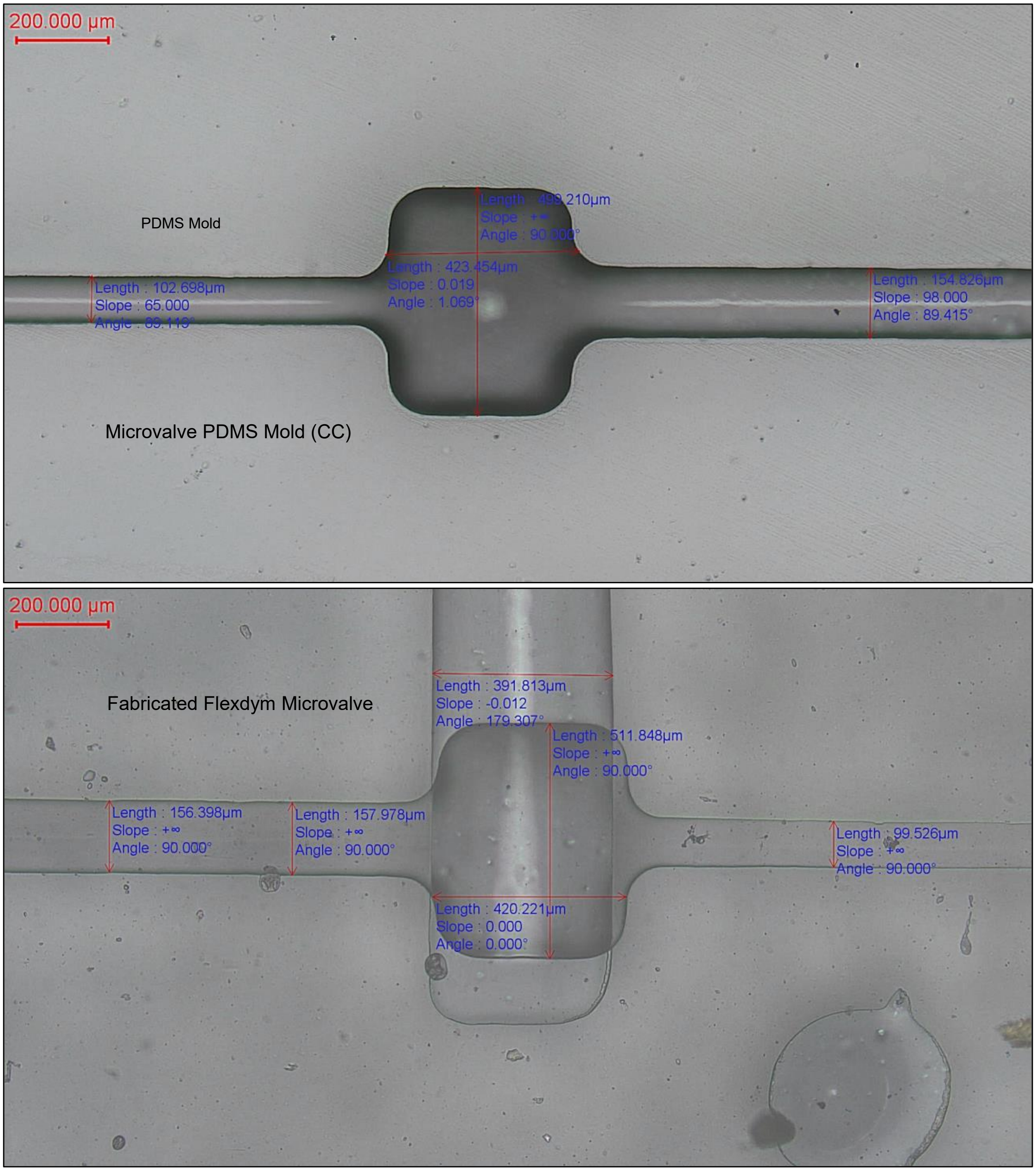

Figure S8. Detail dimensions (width) of actual microvalve PDMS concave mold by thermal air expansion in the top and dimensions of fabricated Flexdym microvalve after thermal replication, alignment and bonding, indicating minimal changes throughout the process, reliable fabrication process and good reproducibility. Scale bars are indicated in the image.

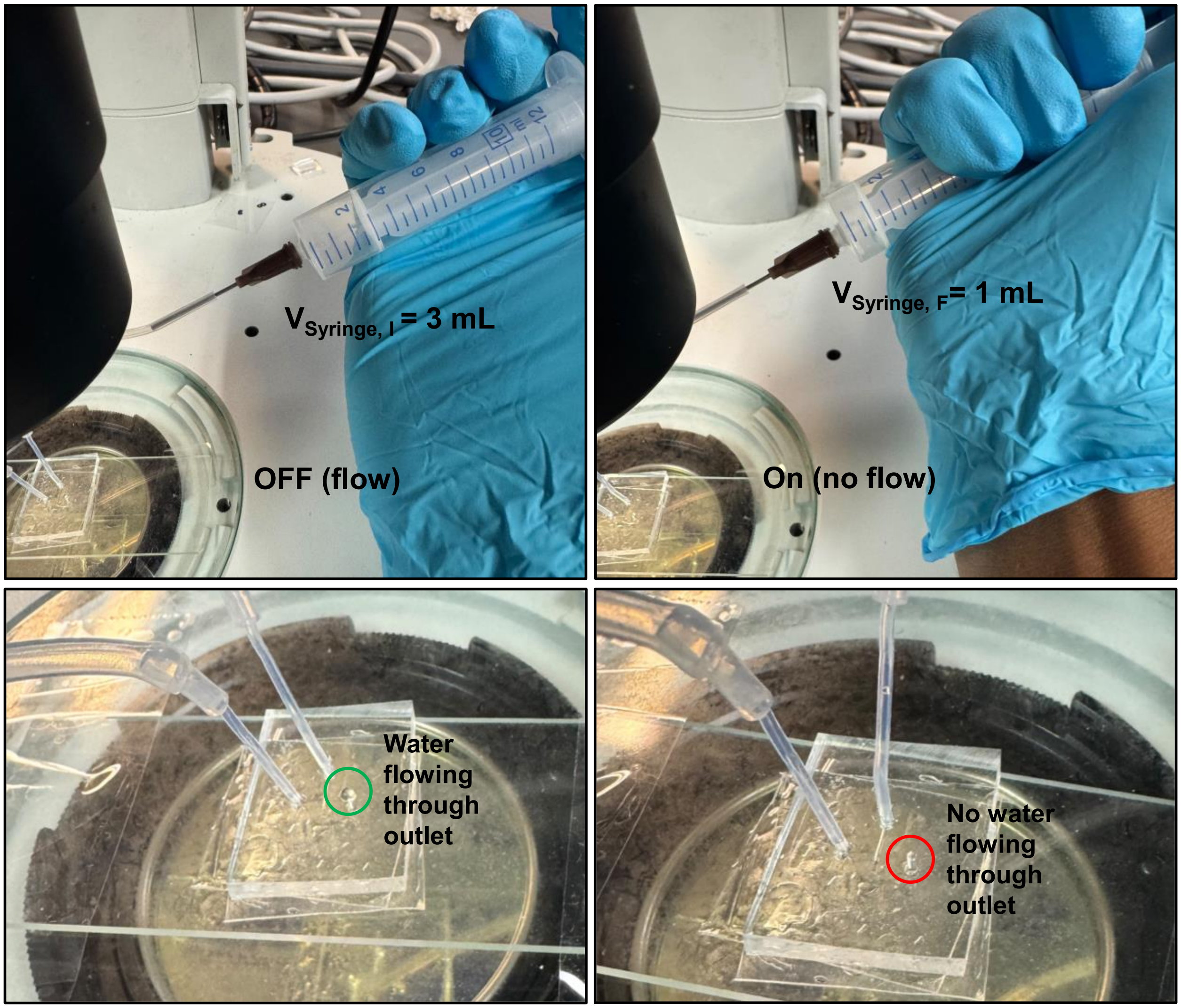

Figure S9. Experimental demonstration of syringe assisted compressed air microvalve actuation. At an initial syringe air volume of 3.0319 mL, the valve remained OFF and water flowed through the outlet in the left two images. Compression of the trapped air to 1.0319 mL actuated the valve to the ON state, completely stopping outlet flow at the right two images.

Table S1. Comparison of channel dimensions across fabricated replicas (C1, Concave PDMS mold; C2, Convex PDMS mold; C3, Flexdym**) A. Channel width**

**Channel**	**Concave PDMS, mean ± SD (µm)**	**Convex PDMS, mean ± SD (µm)**	**Flexdym, mean ± SD (µm)**	**One-way ANOVA**	**Tukey P C2−C1**	**Tukey P C3−C1**	**Tukey P C3−C2**
37 µm	36.352 ± 1.304 (*n*=4)	35.950 ± 2.360 (*n*=4)	38.326 ± 0.779 (*n*=4)	F (2,9) = 2.47; P = 0.139	0.935	0.248	0.150
100 µm	97.580 ± 5.530 (*n*=5)	100.805 ± 1.315 (*n*=5)	100.489 ± 1.797 (*n*=5)	F (2,12) = 1.33; P=0.300	0.334	0.404	0.988
122 µm	121.560 ± 2.940 (*n*=4)	122.868 ± 1.512 (*n*=4)	124.830 ± 2.240 (*n*=4)	F (2,9) = 2.03; P = 0.187	0.712	0.167	0.482
249 µm	249.590 ± 0.896 (*n*=3)	251.191 ± 1.590 (*n*=3)	253.230 ± 2.020 (*n*=3)	F (2,6) = 4.04; P = 0.077	0.471	0.067	0.320

B. Channel height

**Channel**	**Concave PDMS, mean ± SD (µm)**	**Convex PDMS, mean ± SD (µm)**	**Flexdym, mean ± SD (µm)**	**One-way ANOVA**	**Tukey P C2−C1**	**Tukey P C3−C1**	**Tukey P C3−C2**
37 µm	7.263 ± 0.403 (*n*=8)	6.918 ± 0.504 (*n*=8)	7.074 ± 0.328 (*n*=8)	F (2,21) = 1.37; P = 0.277	0.247	0.644	0.738
100 µm	24.208 ± 0.742 (*n*=4)	24.573 ± 0.123 (*n*=4)	24.230 ± 0.966 (*n*=4)	F (2,9) = 0.33; P = 0.724	0.752	0.999	0.778
122 µm	25.738 ± 2.174 (*n*=8)	25.775 ± 1.887 (*n*=8)	26.350 ± 2.470 (*n*=8)	F (2,21) = 0.20; P=0.823	0.999	0.843	0.860
249 µm	62.720 ± 7.630 (*n*=4)	60.200 ± 5.900 (*n*=4)	58.630 ± 4.950 (*n*=4)	F (2,9) = 0.44; P = 0.659	0.839	0.639	0.933

Note. Data are presented as mean ± standard deviation (SD). C1, concave PDMS mold; C2, convex PDMS mold; C3, Flexdym replicas. For each channel dimension, groups were compared using one-way ANOVA followed by Tukey's post hoc multiple-comparison test. $P<0.05$ was considered statistically significant. No overall ANOVA or Tukey pairwise comparison reached statistical significance.

COMSOL Multiphysics Simulation of Water-Driven Air Compression

A transient two-dimensional model was developed in COMSOL Multiphysics 6.0 using the Laminar Two-Phase Flow, Moving Mesh Multiphysics interface. This interface couples the Laminar Flow equations with a Moving Mesh deforming-domain formulation, solves for fluid velocity and pressure, and tracks the air-water interface through deformation of the computational mesh.

The computational geometry consisted of a closed rectangular chamber initially occupied by water in the lower region and trapped air in the upper region, with the phases separated by a horizontal fluid-fluid interface. Water entered through the lower-left inlet at a constant volumetric flow rate of $Q = 200\ \mu\mathrm{L}\,\mathrm{min}^{-1}$. The remaining portion of the left boundary was assigned to a symmetry condition, whereas the top, bottom, and right boundaries were treated as walls. No outlet was selected because the incoming water displaced the interface and continuously compressed the trapped air.

Within each phase i ($i = w$ for water and $i = g$ for air), COMSOL solved the transient conservation of mass and momentum equations in the deformed spatial frame:

$$\frac{\partial \rho_i}{\partial t} + \nabla \cdot (\rho_i \mathrm{u}_i) = 0$$

$$\rho_i \left[\frac{\partial \mathrm{u}_i}{\partial t} + (\mathrm{u}_i \cdot \nabla)\mathrm{u}_i\right] = \nabla \cdot (-p_i \mathrm{I} + \tau_i) + \mathrm{F}_i$$

where ρ_i, u_i, p_i, and F_i denote density, velocity, pressure, and the body-force vector, respectively. For a Newtonian fluid, the viscous stress tensor was written as

$$\tau_i = \mu_i[\nabla \mathrm{u}_i + (\nabla \mathrm{u}_i)^T] - \frac{2}{3}\mu_i(\nabla \cdot \mathrm{u}_i)\mathrm{I}$$

where μ_i is the dynamic viscosity. Gravity was neglected.

Water was modeled as an incompressible Newtonian liquid. To represent compression of the sealed gas volume, air was modeled using the Compressible flow (Ma < 0.3) formulation with pressure-dependent density. Under an isothermal ideal-gas assumption,

$$\rho_g = \frac{p_{A,g} M_g}{R\mathrm{T}}$$

where M_g is the molar mass of air, R is the universal gas constant, and T is the ambient temperature in K. COMSOL relates absolute pressure to the solved pressure variable according to

$$p_{A,g} = p_g + p_{\mathrm{ref}}$$

where p_{g} is the air pressure and p_{ref} is the reference pressure, used default.

The initial air-water boundary was defined using the Fluid-Fluid Interface feature. No interphase mass transfer was assumed. Accordingly, velocity continuity and the kinematic interface condition were imposed as

$$\mathrm{u}_w = \mathrm{u}_g$$

$$\mathrm{u}_m \cdot \mathrm{n} = \mathrm{u} \cdot \mathrm{n}$$

$$(\mathrm{T}_g - \mathrm{T}_w)\mathrm{n} = \gamma\kappa\mathrm{n} + \nabla_s\gamma$$

where $\mathrm{T}_i = -p_i\mathrm{I} + \tau_i$, γ is the surface tension in the interface , κ is the interface curvature, and ∇_s is the surface-gradient operator. For constant surface tension, $\nabla_s\gamma = 0$. u_m is the moving mesh or interface velocity.

The entire computational domain was defined as a deforming domain using the Moving Mesh interface to track displacement of the air–water interface. The domain was discretized using a free triangular mesh with the COMSOL predefined Fine element size for fluid-flow calculations. A time-dependent study was performed over 0–8 s, and the coupled Multiphysics equations were solved using the PARDISO direct solver. Water was introduced through a fully developed laminar-flow inlet at $Q = 200\ \mu\mathrm{L\,min}^{-1}$.
